# Conjugated Microporous Polymers‐Based Catalytic Membranes with Hierarchical Channels for High‐Throughput Removal of Micropollutants

**DOI:** 10.1002/advs.202401966

**Published:** 2024-06-03

**Authors:** Jiaqiang Li, Wei Lyu, Xuejin Mi, Cheng Qian, Yanbiao Liu, Junrong Yu, Richard B. Kaner, Yaozu Liao

**Affiliations:** ^1^ State Key Laboratory for Modification of Chemical Fibers and Polymer Materials College of Materials Science and Engineering Donghua University Shanghai 201620 China; ^2^ Textile Pollution Controlling Engineering Center of Ministry of Environmental Protection College of Environmental Science and Engineering Donghua University Shanghai 201620 China; ^3^ Department of Chemistry and Biochemistry Department of Materials Science and Engineering and the California NanoSystems Institute University of California Los Angeles CA 90095 USA

**Keywords:** bioinspired hierarchically porous networks, catalytic membrane, conjugated microporous polymer, high‐throughput removal of micropollutants, water treatment

## Abstract

Engineering a catalytic membrane capable of efficiently removing emerging organic microcontaminants under ultrahigh flux conditions is of significance for water purification. Herein, drawing inspiration from the functional attributes of lymphatic vessels involved in immunosurveillance and fluid transport with minimal energy consumption, a novel hierarchical porous catalytic membrane is engineered. This membrane, based on an innovative nitrogen‐rich conjugated microporous polymer (polytripheneamine, PTPA), is synthesized using an electrospinning coupled in situ polymerization approach. The resulting bioinspired membrane with hierarchical channels comprises a thin layer (≈1.7 µm) of crosslinked PTPA nanoparticles covering the interconnected electrospun nanofibers. This unique design creates an intrinsic microporous angstrom‐confined system capable of activating peroxymonosulfate (PMS) to generate 98.7% singlet oxygen (^1^O_2_), enabling durable and highly efficient degradation of microcontaminants. Additionally, the presence of a thin layer of mesoporous structure between PTPA nanoparticles and macroporous channels within the interwoven nanofibers enhances mass transfer efficiency and facilitates high flux rates. Notably, the prepared hierarchical porous organic catalytic membrane demonstrates enduring high‐efficiency degradation performance with a superior permeance (>95% and >2500 L m^−2^ h^−1^ bar^−1^) sustained over 100 h. This work introduces an innovative pathway for the design of high‐performance catalytic membranes for the removal of emerging organic microcontaminants.

## Graphical abstract

Inspired by the functional attributions of lymphatic vessels in immunosurveillance and fluid transport with minimal energy consumption, a novel hierarchical porous polymer catalytic membrane, based on the innovative nitrogen‐rich conjugated microporous polymers, is engineered for ultrafast, highly efficient, and long‐lasting removal of emerging organic micropollutants from water under ultrahigh flux conditions.

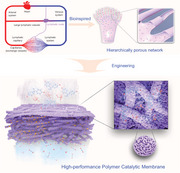

## 1 Introduction

In the contemporary era, the persistence and accumulation of emerging trace organic pollutants within the aquatic ecosystem, encompassing endocrine‐disrupting chemicals, antibiotics, pharmaceuticals, personal care products, and pesticides, pose a substantial threat to both human health and environmental systems.^[^ ^1^ ^]^ Nevertheless, these minute contaminants have been proved to be formidable challenges when attempting their removal from water using conventional biological methods, owing to their low concentrations and resistance to the majority of microorganisms.^[^ ^2^ ^]^

The integration of catalytic membrane systems, combining advanced oxidation processes (AOPs) with membrane separation, has emerged as a cutting‐edge water purification technology that has garnered significant attention for mitigating the presence of these microcontaminants.^[^ ^3^ ^]^ The appeal of this approach primarily emanates from its ability to confer the catalyst with facile recyclability and enhanced mass transfer, as dictated by Fick's law, while simultaneously endowing the membrane with self‐cleaning capabilities.^[^ ^4^ ^]^

In the realm of catalytic materials, inorganic catalysts such as metal‐based and carbon‐based materials have garnered favor due to their high catalytic activity. Nevertheless, they encounter challenges in fabricating thin, dense active layers with controllable porous structures and tunnels to achieve high flux. Moreover, it should be noted that metal‐based catalysts may suffer from the inevitable metal leaching. In contrast, polymer materials, owing to their molecular design versatility and processability, could offer a more facile avenue for the design and construction of organic membranes that feature both nanoscale confinement spaces and high flux in a controllable manner. However, polymer‐based catalytic membranes face with issues like relatively low degradation efficiency, poor stability, and a short service life, stemming from the vulnerability to be attacked by highly reactive oxygen species (ROS) like SO_4_^•—^ and •OH.^[^ ^3b^ ^]^ A promising strategy to avoid self‐degradation in the membrane involves the precise generation of singlet oxygen (^1^O_2_), a moderately reactive electrophile capable of selectively degrading electron‐rich organic pollutants.^[^ ^5^ ^]^ Nevertheless, the challenge lies in the fact that free radicals typically accompany the generation of ^1^O_2_ in advanced oxidation processes (AOPs).^[^ ^6^ ^]^ Consequently, the creation of a long‐lasting, ultrafast catalytic membrane with exceptional degradation efficiency (exceeding 95%) under an ultrahigh flux of greater than 2500 L m^−2^ h^−1^ bar^−1^ remains a formidable task. Recently, porous organic polymers (POPs) such as hypercrosslinked polymers (HCPs), covalent organic frameworks (COFs) and conjugated microporous polymers (CMPs) have been broadly utilized in the treatment and separation of emerging trace organic pollutants owing to their uniform pore sizes, high porosity and molecular designability.^[^ ^7^ ^]^ Especially membranes made of POPs have achieved remarkable results in nanofiltration and micropollutant separation.^[^ ^8^ ^]^ However, the trade‐off between membrane permeability and selectivity, along with the persistent issue of membrane pollution, continues to pose significant challenges for these synthetic non‐catalytic membranes.

**Figure 1:**
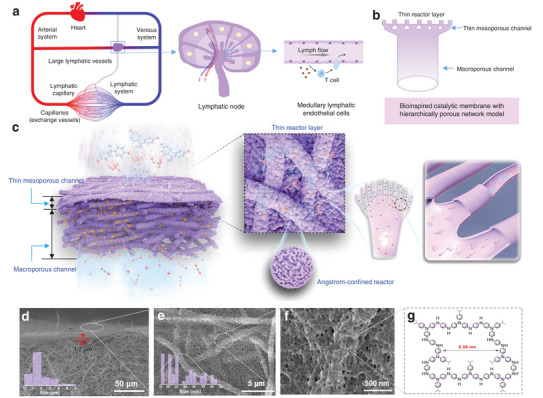
Biological inspiration and schematic of bioinspired catalytic membrane for micropollutants’ removal. a) Hierarchical networks of blood and lymphatic vessels and tissue immunosurveillance of thin lymphatic endothelial cells. b) Inspired hierarchically porous network model for a catalytic membrane. c) Schematic illustration of the bioinspired catalytic membrane built on PMS‐based AOP. It contains a thin layer of micropores for micropollutants’ degradation on the top, and branched mesopores and micropores for increasing permeability. d–f) SEM images of the as‐prepared bioinspired catalytic membrane containing macropores and mesopores under different magnifications. g) Proposed chemical structure of PTPA with intrinsic micropores.

Recent progress in the inspiration of biological structure and function allows for the construction of membrane with high performance.^[^ ^9^ ^]^ Here, we report a CMP catalytic membrane with bioinspired hierarchical porous networks with near 100% ^1^O_2_ generation, enabling the long‐term and ultrafast degradation of organic micropollutants under ultrahigh flux conditions. The intricate vascular networks of blood and lymphatic vessels play pivotal roles in maintaining bodily hemostasis and defending against various diseases. These networks exhibit a largely fractal geometric organization that facilitates high fluid uptake and efficient mass transfer with minimal energy consumption.^[^ ^10^ ^]^ Within lymphatic nodes, the branched sinus systems derived from lymphatic vessels are lined with a thin layer of discontinuously connected lymphatic endothelial cells, representing a distinct endothelial cell lineage characterized by specific transcriptional and metabolic programs for tissue immunosurveillance (**Figure** **1**a).^[^ ^10^ ^]^ The discontinuous junctions between cells increase permeability and facilitates the capture of interstitial components and the transmigration of immune cells. Inspired by the functional attributes of this biological system, which features hierarchical channels lined with thin‐layer reactors (Figure 1b), we have developed an artificial bioinspired catalytic membrane based on CMPs (Figure 1c).

This innovative bioinspired catalytic membrane relies on a peroxymonosulfate (PMS)‐based AOP that generates ^1^O_2_ through a non‐radical pathway by designing catalysts with electron‐capture capabilities.^[^ ^11^ ^]^ Polytriphenylamine (PTPA), a nitrogen‐rich CMP resembling polyaniline (PANI) with a primary intrinsic micropore size of 0.58 nm,^[^ ^12^ ^]^ serves as the microporous reactor responsible for generating near 100% ^1^O_2_ within the membrane, owing to its angstrom‐confinement effect. As illustrated in Figure 1d‐g, the bioinspired catalytic membrane comprises a thin layer (≈1.7 µm) of PTPA particles with an average mesopore size of ≈27 nm situated between these connected particles at the apex of interconnected electrospun nanofibers with an average macropore size of ≈1.7 µm. This membrane was fabricated using an electrospinning‐coupled in situ polymerization approach. The designed bioinspired membrane exhibited robust, high‐efficiency degradation performance (*K* = 1430.9 min^−1^, with over 95.0% degradation efficiency sustained for at least 100 hours) while maintaining superior permeance (greater than 2500 L m^−2^ h^−1^ bar^−1^, 4 times higher than the highest value of current reported catalytic membrane). This catalytic performance surpassed that of numerous state‐of‐the‐art catalytic membranes. Our research opens up new possibilities for the development of high‐performance polymer catalytic membranes for the continuous removal of organic micropollutants and broadens the applicability of CMPs as efficient membrane‐based catalysts.

## 2 Result and Discussion

### 2.1 Synthesis, Characterization, and Catalytic Performance of PTPA

**Figure 2:**
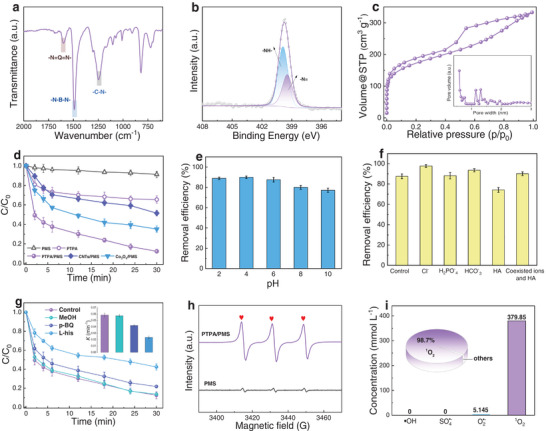
Characterization and catalytic performance of PTPA. a) FT‐IR spectra, b) N 1s XPS spectra, c) N_2_ adsorption–desorption isotherms (77 K) of PTPA, with the inset showing the pore size distribution. d) Removal efficiency of different reaction systems. e) Initial pH and f) effect of different anions, HA, and coexisted ions and HA on the BPA removal efficiency. g) Comparison of BPA removal efficiency and pseudo‐first‐order rate constant under different radical scavengers. For d–g) the statistics are based on a sample size of *n*  =  3, and data are presented as mean ± s.d. h) EPR spectra of different systems using TEMP as the trapping agent. i) Quantitative determination of ROS. (Conditions: [Catalyst] = 0.20 g L^−1^, [BPA] = 25 mg L^−1^, [PMS] = 1.0 mmol L^−1^, *T* = 25 °C, initial pH 6.0, [Cl^‐^] = 15 mmol L^−1^, [H_2_PO_4_^‐^] = 15 mmol L^−1^, [HCO_3_^‐^] = 15 mmol L^−1^ and [HA] = 15 mg L^−1^, [MeOH] = 2 mol L^−1^, [p‐BQ] = 5 mmol L^−1^, [L‐his] = 5 mmol L^−1^, TEMP] = 50 mmol L^−1^).

PTPA was synthesized by a Buchwald–Hartwig (BH) cross‐coupling reaction with a Bristol–Xi'an Jiaotong (BXJ) method (Scheme S1, Supporting Information).^[^ ^13^ ^]^ FT‐IR spectra of PTPA exhibits three characteristic stretching vibrations located at 1599 (*ν*_C__═__C_ for quinoid rings), 1490 (*ν*_C__═__C_ for benzenoid rings) and 1245 cm^−1^ (─C─N─) (**Figure** **2**a), indicating its PANI‐like structure.^[^ ^13^, ^14^ ^]^^13^C CP/MAS NMR spectra of PTPA shown in Figure S1 (Supporting Information) further support the successful formation of this amine‐linked conjugated polymer network, with two main peaks located at ≈144 (C─N) and ≈127 ppm (C═C) observed. The elemental composition of the as‐prepared PTPA was characterized by XPS spectra and EA (Table S1, Supporting Information). In Figure 2b and Figure S2 (Supporting Information), two characteristic peaks attributing to C─C/C─H (284.6 ± 0.1 eV) and C─N/C═N bonds (≈285.5 ± 0.1 eV) were observed in the C 1s XPS spectra,^[^ ^15^ ^]^ while two characteristic peaks ascribed to ─NH─ (399.6 ± 0.1 eV) and ─N═ bonds (398.8 ± 0.3 eV) appeared in the N 1s XPS spectra of PTPA.^[^ ^15^ ^]^ Moreover, TGA indicates that PTPA exhibits relatively good thermostability due to its cross‐linked conjugated network (Figure S3, Supporting Information). SEM and TEM analyses show that PTPA possesses a crosslinked nanoparticle morphology (Figure S4, Supporting Information). N_2_ adsorption–desorption isotherm analyses of PTPA shown in Figure 2c demonstrates that PTPA has a typical type‐IV isotherm reflecting its unique micropore features.^[^ ^16^ ^]^ This is supported by pore size distribution analyses (inset in Figure 2c), where micropores with pore diameters centered at 0.58 nm and 1.14–1.3 nm, derived from nonlocal density function theory (NLDFT) method, were observed. Due to its high microporosity, a BET surface area (*S*_BET_) of 684 m^2^ g^−1^ was obtained (Table S2, Supporting Information).

In order to reflect the complete intrinsic catalytic activity of catalyst, PTPA active catalyst, rather than the whole membrane, was adopted to investigate a variety of parameters on the catalytic performance in the batch reaction system. Bisphenol A (BPA, an endocrine disrupting chemical with poor biodegradability)^[^ ^17^ ^]^ was used as a model pollutant. As presented in Figure 2d and Figure S5 (Supporting Information), it can be noted that the contributions of adsorption on the removed BPA is small amount. Moreover, PTPA/PMS exhibits a significantly higher BPA degradation rate with the value of a pseudo‐first‐order constant (*K*) = 5.58 × 10^−2^ min^−1^, which is 1.8 and 3.5 times higher than that of Co_3_O_4_ (a standard metal catalyst, 3.02 × 10^−2^ min^−1^) and CNTs (a standard metal‐free carbon catalyst, 1.59 × 10^−2^ min^−1^), respectively (Figure S6, Supporting Information). The better degradation performance of PTPA on BPA may be attributed to its higher decomposition efficiency of PMS by PTPA (Figure S7, Supporting Information).^[^ ^18^ ^]^ Meanwhile, more than 87.3% of BPA was removed within 30 min in the PTPA/PMS system. Notably, further extending reaction time to 120 min could achieve a 98.4% remove efficiency (Figure S8a, Supporting Information). An almost 100% removal efficiency was achieved at 60 min when the PTPA dosage was 0.3 g L^−1^ and the initial concentration of BPA was 5 ppm (Figure S8b, Supporting Information). This indicates that PTPA is superior to some common heterogeneous metal catalysts and competitive with recently reported best metal‐free carbon catalysts (Table S3, Supporting Information).

The versatility of PTPA/PMS under different conditions is of great importance. We therefore evaluated the impact of the initial pH, anion (Cl^‐^, HCO_3_^‐^, H_2_PO_4_^‐^) and humic acid (HA) (a component among natural organic matter (NOM) with 1–20 mg L^−1^ existing in rivers or groundwater^[^ ^19^ ^]^) on the degradation of BPA (Figure 2e,f and Figure S9 in Supporting Information). PTPA is effective in degrading BPA over a much wider initial pH ranging from 2.0 to 10.0. It can be observed from Figure S10 (Supporting Information) that the final pH values were kept around 3.0 ± 0.3 when the initial pH ranged from 4.0 to 8.0 likely due to the buffering ability of the catalyst.^[^ ^20^ ^]^ Therefore, there was almost no significantly difference in the degradation rate (Figure 2e). In addition, there was no obvious influence on the degradation performance of BPA at different pHs (maintained with a buffer solution), further illustrating the good catalytic ability of the PTPA/PMS system for BPA degradation over a wide pH range (Figure S11, Supporting Information).^[^ ^21^ ^]^ Figure 2f and Figure S9 (Supporting Information) and Table S4 (Supporting Information) show the effect of coexisting anions and HA on the BPA degradation. As can be seen in Figure S9c (Supporting Information), HCO_3_^‐^ played a promoting effect on BPA degradation, with the *K* increasing from 5.58 to 7.16 × 10^−2^ min^−1^ and the degradation rate increasing from 87.3 to 93.5% at a HCO_3_^‐^ concentration of 15 mmol L^−1^. The possible reason is because HCO_3_^‐^ can withstand the acidification of PMS and generate a good platform for PTPA to fully exert its catalytic function (Figure S12, Supporting Information).^[^ ^22^ ^]^ Comparable, H_2_PO_4_^‐^ showed no effect on the degradation of BPA (Figure S9b, Supporting Information). Notably, the enhanced removal performance (degradation rate increased from 87.3 to 97.7% at a Cl^‐^ concentration of 15 mmol L^−1^) in the presence of Cl^‐^ might be due to the Cl^•^ that is generated from the reaction between Cl^‐^ and O_2_^• ‐^ (Figure S9a, Supporting Information).^[^ ^23^ ^]^ As shown in Figure S13 (Supporting Information), only EtOH could not change the degradation efficiency, but the co‐existence of EtOH and Cl^‐^ did induce a suppression of the enhanced removal performance in the system containing Cl^‐^. While a slight decrease in the removal efficiency (degradation rate decreased from 87.3% to 74.2%) observed in the presence of HA with a high concentration (i.e., 15 mg L^−1^) may have resulted from the fact that HA could block the active sites of PTPA via strong π–π stacking and thus prevent the interaction of PTPA with PMS (Figure S9d, Supporting Information).^[^ ^22^ ^]^ The obtained slight promotion of degradation efficiency in the solution containing coexisted ions and HA further implies a nonradical reaction pathway for BPA degradation, indicating that the PTPA/PMS system has good interference immunity and high removal performance under complex conditions (Figure 2f).^[^ ^24^ ^]^ The effect of other conditions on the degradation of BPA, including the initial BPA concentration, PTPA dosage, PMS concentration and reaction temperature, were also evaluated as shown in Figures S14 and S15 (Supporting Information).

### 2.2 Angstrom‐Confinement Catalytic Mechanism of PTPA

We then identified the generated ROS by using radical scavenger experiments. As shown in Figure 2g, no obvious inhibition was observed on the BPA removal after adding methanol, indicating that the contribution of SO_4_^• ‐^ and ^•^OH is negligible. A ≈20% reduced kinetic constant was noted after adding *p*‐benzoquinone (p‐BQ), suggesting O_2_^•‐^ existed in the system. Remarkably, a significant negative influence on the BPA removal was observed when adding L‐histidine (L‐his), furfuryl alcohol (FFA) or β‐carotene (Figure S16, Supporting Information), indicating that ^1^O_2_ was the main reactive species in the PTPA/PMS system. EPR spectra (Figure 2h) further supports this analysis, where a strong triplet peak signal characteristic (1:1:1) of 2,2,6,6‐tetramethyl‐2‐piperidinol (TEMP) was observed. Moreover, by using 5,5‐dimethyl‐pyroline *N*‐oxide (DMPO) as the trapping agent, six peaks characteristic of DMPO‐O_2_^• ‐^ were detected, while the characteristic peaks for DMPO‐^•^OH and DMPO‐SO_4_^• ‐^ were not detected (Figure S17, Supporting Information). To further quantify the ROS produced in the PTPA/PMS system, the concentrations of ROS were determined by using the EPR method on a quantitative basis,^[^ ^25^ ^]^ where the proportion of ^1^O_2_ in the ROS generated was 98.7% (Figure 2i). The ≈100% ^1^O_2_ allows PTPA to possess excellent stability (Figure S18, Supporting Information). For comparison, the detected TEMP signal was relatively weak in the PANI/PMS system (Figure S19, Supporting Information). Moreover, the observed negative influence on the BPA removal upon adding p‐BQ (Figure S20, Supporting Information) demonstrates that O_2_^• ‐^ was the predominant reactive species in the PANI/PMS system. Notably, both PANI and PTPA showed similar chemical structures and exhibited the same N types (amine N and imine N), in which PANI possessed a higher content of N from XPS and EA analyses (Figure S21, Supporting Information). The above results therefore indicate that the confinement of N atoms indeed changed the PMS reaction pathway, rather than N contents and types. Similar phenomenon was also observed for PTPA‐0 without micropores (*S*_BET_ is 15 m^2^ g^−1^), in which O_2_^• ‐^ was the main reactive species (Figure S22, Supporting Information). This further confirms the vitality of the confined voids in the generation of ^1^O_2_. NaF salt also did not play a role in the generation of ^1^O_2_ (Figure S23, Supporting Information).

**Figure 3:**
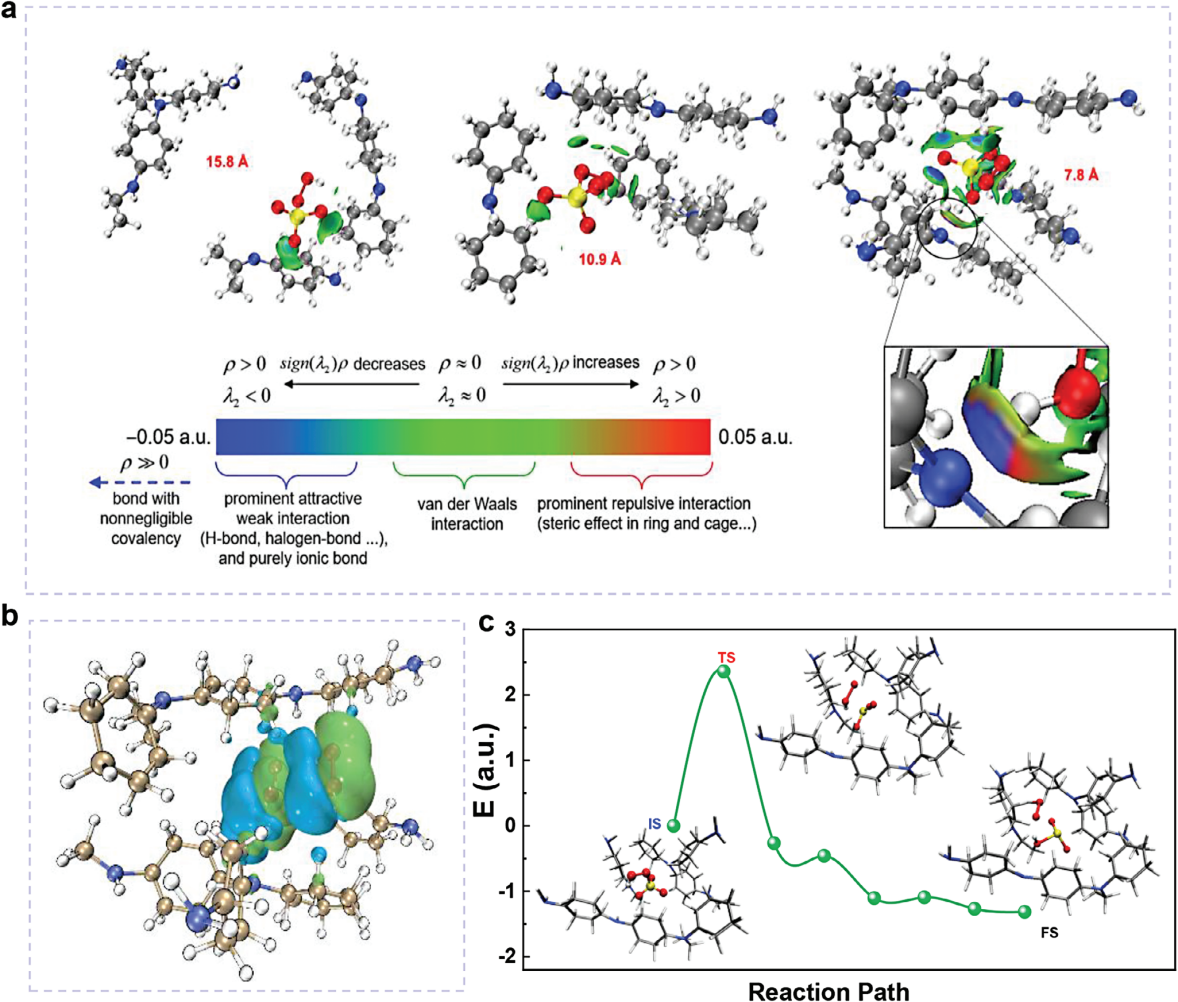
Mechanism of angstrom‐confinement catalysis of PTPA. a) Weak interaction regions visualized with a δ_g_^inter^ isovalue of 0.005 a.u. in the IGMH analysis for PTPA and PMS under different pore size. b) Electronic density difference for PMS intercalating into PTPA under the angstrom‐confinement of 7.8 Å; green and blue represent electron accumulation and depletion, respectively. c) Energy profile during the dissociation of PMS under the angstrom‐confinement of 7.8 Å.

Control experiments in which nitrogen and oxygen gases were used to purge the reactor of oxygen was first carried out to determine the contribution of dissolved oxygen on the production of ^1^O_2_. As shown in Figure S24 (Supporting Information), a slight decrease (7.13%) in degradation efficiency was observed in a nitrogen gas atmosphere and almost no influence on BPA degradation was noted in an oxygen gas atmosphere. The above results suggest that a small part of ^1^O_2_ originated from dissolved oxygen in the reaction solution. An EPR experiment was then performed with the addition of p‐BQ to analyze the contribution from O_2_^• ‐^. A slightly weakened intensity of the ^1^O_2_ EPR signal after adding p‐BQ indicates that O_2_^• ‐^ is an intermediate for the generation of a small part of ^1^O_2_ (Figure S25, Supporting Information). Therefore, we deduce that the majority of ^1^O_2_ was related to the confined voids, which was then studied by using density functional theory (DFT) computations. The amorphous polymer models were generated using an oligomer construction method (Figure S26, Supporting Information).^[^ ^26^ ^]^ Figure S27a (Supporting Information) shows the void space architecture of the PTPA model, with N_2_ as the probe molecule. The surface area for the 0.8 g cm^−3^ PTPA model was calculated at around 729 m^2^ g^−1^ by using a Monte Carlo code,^[^ ^27^ ^]^ which agrees well with the experimental data (685 m^2^ g^−1^). The calculated pore size was centered at 4.08 and 6.37 Å (Figure S27b, Supporting Information), which is also close to the experimental data. Three different confined fragments with pore sizes of 15.8, 10.9, and 7.8 Å (van der Waals diameters of 7.5, 4.9, and 3.3 Å,^[^ ^28^ ^]^ respectively) were selected to study the role of pore size on PMS activation (Figure S28, Supporting Information). Notably, the actual diameter of these micropores may be larger than the calculated van der Waals diameter due to the irregular nature of these pores, and herein we focus on the effect of gradually decreasing pore size. As shown in Figure S30 (Supporting Information), the interaction energy (*E*_int_) of PMS increased from −0.54 to −0.70 eV when the pore size decreased from 15.8 Å (van der Waals diameter of 7.5 Å) to 10.9 Å (van der Waals diameter of 4.9 Å). Moreover, the length of the S─O bond in PMS increased with decreasing pore size (Figure S30, Supporting Information). When the pore size was around 7.8 Å (van der Waals diameter of 3.3 Å), PMS dissociation occurred through the cleavage of the S─O bond with the subsequent generation of ^1^O_2_, with an *E*_int_ of −0.63 eV achieved. Interestingly, as the Hirshfeld partition of molecular density (IGMH) analysis (**Figure** **3**a) indicates, the observed blue color on the IGMH δ_g_^inter^ isosurface of the O atom of HSO_3_^‐^ and the H atom of PTPA suggests the existence of attractive interactions between HSO_3_^‐^ and PTPA, which are absent in the pore sizes of 15.8 and 10.9 Å. In the unconfined system using linear PANI, PMS is mainly activated via the redox pathway by attracting PMS on amine N, in which PMS gains electrons to produce radicals (^•^OH and SO_4_^• ‐^) or loses electrons to produce O_2_^• ‐^ (Figure S20, Supporting Information).^[^ ^23^, ^24^, ^25^, ^26^, ^27^, ^28^, ^29^ ^]^ While in this confined system, a red color shown on the IGMH δ_g_^inter^ isosurface of the N atom of PTPA suggests the existence of repulsive interactions resulting from steric effects, which may change the active sites from N atoms of amine group to H atoms of benzenoid ring and led to the formation of ^1^O_2_. Electron density difference (EDD) analysis (Figure 3b) shows that the large redistributions of electronic densities from the O atom of decomposed HSO_3_^‐^ to the H atom of the benzenoid ring, indicating the possible existence of hydrogen bonds between PMS and PTPA conjugated skeletons in this angstrom‐confinement system. Therefore, we deduce that in such an angstrom‐confined space of PTPA, PMS self‐decomposes into HSO_3_^‐^ and ^1^O_2_ species with the cleavage of S─O bond at a more thermodynamically stable state,^[^ ^30^ ^]^ instead of gaining or losing electrons involved in the redox pathway that occurs in the unconfined system. Climbing image‐nudged elastic bond (CINEB) was then used to further search the transition state (TS) of the dissociated PMS in the final state (FS) under the confinement of 7.8 Å. As shown in Figure 3c, a TS (+2.36 eV) with a longer distance between S and O (3.40 vs 2.05 Å the of initial state (IS)) and a relatively decreased O─O bond length (1.36 vs 1.41 Å of IS) was found. It indicates the occurrence of dissociation through the cleavage of S─O, which is similar to the mechanism of PMS dissociation in Co‐TiO*_x_* membranes.^[^ ^30^ ^]^ Moreover, the negative free energy of −1.31 eV demonstrates that this process is spontaneous and thermodynamically favorable. Notably, those simulated calculations provide us with a possible formation mechanism due to the irregular pore sizes of PTPA. The possible degradation mechanism and degradation route are proposed and shown in Figures S31–S34 (Supporting Information).

### 2.3 Controllable Fabrication and Performance of Hierarchical Porous Catalytic Membranes

**Figure 4:**
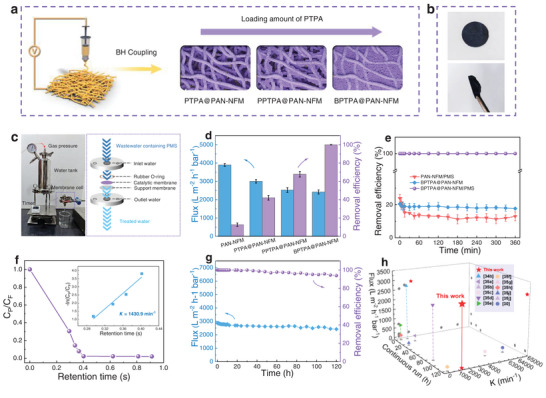
Fabrication and removal performance of catalytic membranes. a) Fabrication of catalytic membranes. b) The photograph of BPTPA@PAN‐NFM. c) Photograph of the experimental setup, with a schematic representation of the configuration of the flow‐through device (right). d) Permeance and efficiency of BPA removal of catalytical membranes. For c,d) the statistics are based on a sample size of n  =  3, and data are presented as mean ± s.d. e) Removal efficiency of BPA in different reaction systems. f) Normalized concentration of BPA (*C*_permeate_/*C*_feed_) versus membrane retention time with inset shown the pseudo‐first‐order kinetic. g) Stability test of flux and removal efficiency under the pressure‐dependent continuous‐flow operation. h) Comparison of flux, continuous running time and the pseudo‐first‐order rate constant *K* values. (Conditions: pressure = 0.2 bar, [PMS] = 1.0 mmol L^−1^, [BPA] = 2 mg L^−1^, *T* = 25 °C, initial pH = 6.0).

Different PTPA‐based hierarchical porous catalytical membranes, including a PTPA coated PAN nanofiber mat (PTPA@PAN‐NFM), a partially crosslinked PTPA nanoparticle coated PAN nanofiber mat (PPTPA@PAN‐NFM) and a bioinspired PTPA thin membrane coated PAN nanofiber mat (BPTPA@PAN‐NFM), were fabricated by controlling the concentration of PTPA on the surface of electrospun PAN‐NFM in a blend electrospinning combined in situ polymerization approach (**Figure** **4**a,b and Figure S38 in Supporting Information). The loading amount of PTPA on PTPA@PAN‐NFM, PPTPA@PAN‐NFM and BPTPA@PAN‐NFM membranes were 0.37, 0.45, and 0.53 mg cm^−2^, respectively. Specifically, a low loading amount leads to a membrane with macropores and micropores on the surface of nanofibers (PTPA@PAN‐NFM). Increasing the loading amount of PTPA yields PPTPA@PAN‐NFM, characterized by a partial PTPA thin layer containing micropores and mesopores on the surface, but with some macropores on the surface still uncovered. As for BPTPA@PAN‐NFM, featuring the highest loading amount of PTPA nanoparticles, a complete crosslinking with generated mesopores forms a thin layer across the entire surface. The catalytic membranes with different areas are shown in Figure S39 (Supporting Information). Currently, mass‐scale preparation of larger area in the industrial scale is constrained by electrospinning technology. However, it should be noted that this challenge is believed to be overcome by optimizing technologies, including receiver size, spinneret structures, and stability,^[^ ^31^ ^]^ which are under investigation. These membranes were sealed in the stainless‐steel filter holder, in which all continuous permeation experiments were performed using BPA (2 mg L^−1^ was selected for membrane catalytic experiments in consideration of practical applications)^[^ ^32^ ^]^ as a target pollutant in a dead‐end filtration mode (Figure 4c). Figure 4d shows the catalytic performance and water flux of these membranes. It can be seen that the removal efficiency of BPTPA@PAN‐NFM increased from 12.7% to 100% after inducing PTPA thin membrane on the surface of PAN‐NFM, and the water flux can remain as high as 2870 L m^−2^ h^−1^ bar^−1^. The synergistic effect of angstrom‐confined catalytic channels, i.e., PTPA's intrinsic micropores, on the entire surface, integrated with short mesoporous channels generated by PTPA nanoparticles and the lower layers of macroporous channels of BPTPA@PAN‐NFM is supposed to the main reason for its high mass transfer and removal performance. In comparison, the removal efficiency of PTPA@PAN‐NFM and PPTPA@PAN‐NFM only reached less than 80%, indicating the critical role of the thin PTPA layer on the surface. The comparison of structural characteristics and removal performance of these membranes is shown in Table S6 (Supporting Information). It can be concluded that the loading amount of the active substance affects the pore size of membrane's surface and the formation of bioinspired hierarchical channels. These factors synergistically impact the removal efficiency and permeability of catalytic membranes, with 100% removal efficiency achieved for BPTPA@PAN‐NFM, featuring with the highest loading amount, a complete thin layer across the entire surface and micropores‐mesopores‐macropores hierarchical channels. In addition, distinguishing from the reported thin‐layer microporous structure, the thin PTPA lay serves as a reactor rather than a sieve or filter,^[^ ^33^ ^]^ which endows the membrane with the functional attributes of lymphatic nodes. Notably, the removal efficiency of BPTPA@PAN‐NFM only achieved around 20% without adding PMS, demonstrating that the contribution from adsorption and size‐sieve separation is marginal (Figure 4e). Moreover, it only takes about 0.41 s to degrade BPA, and the pseudo‐first‐order rate constant *K* of the BPA degradation process is as high as 1430.9 min^−1^ (Figure 4f). Under the pressure‐dependent continuous‐flow operation, the degradation efficiency of BPA in the BPTPA@PAN‐NFM membrane system was maintained at 100.0% within 20 h, and decreased slightly to 95.0% after 100 h (2574 L m^−2^ h^−1^ bar^−1^) and 94.0% after 120 h (2420 L m^−2^ h^−1^ bar^−1^), respectively (Figure 4g). The excellent removal performance can be mainly ascribed to the highly exposed confined active sites of PTPA upon contacting with PMS, allowing for the acceleration of electron transfer owing to the minimal diffusion distance and mass transfer enhancement. More importantly, the water flux through the BPTPA@PAN‐NFM remained at a relatively high level with a slight fluctuation (≈2500 L m^−2^ h^−1^ bar^−1^), indicating a negligible fouling performance during the catalytic filtration. To evaluate the stability of the BPTPA@PAN‐NFM, the chemical structure, morphology and crystalline structure of BPTPA@PAN‐NFM before and after continuous‐flow test (120 h) were analyzed (Figures S40 and S41, Supporting Information). It can be seen that the morphology and crystalline structure of the catalytic membrane remained nearly unchanged, indicating the good stability of the BPTPA@PAN‐NFM. It should be mentioned that the weakening of the peak at 1497 cm^−1^ and the enhancement of the peak at 1454 cm^−1^ in FT‐IR spectra may be attributed to the transfer of quinoid ring into benzene ring after 120 h (Figure S40, Supporting Information). This can be resulted from the generation of O_2_^• ‐^ by oxidizing PMS during the prolonged operation, which is consistent with the mechanism above (Equation S5, Supporting Information). Nevertheless, the reduction of ─NR_2_^+^─/─NR‐from ═NR^+^─/═N─ is a redox process of PTPA in different oxidation state, which is a reversible process.^[^ ^14^ ^]^ Moreover, no material leaked on the support membrane after continuous‐flow degradation experiment further demonstrated the stability of the catalytic membrane (Figure S42, Supporting Information).^[^ ^4a^ ^]^ Apart from BPA, the removal efficiency of other electron‐rich organic pollutants with suitable size like phenol and hydroquinone (HQ) can also achieve ≈100.0%. While for organic pollutants with larger size than the main microporous size (0.58 nm) of PTPA, including tetracycline (TC), and dyes (reactive black 5 (RB5), acid red G (ARG), rhodamine B (RhB), and methylene blue (MB)), more than 80% of degradation efficiency were obtained (Figure S43, Supporting Information). To simulate the natural environment, the catalytic performance of BPA in actual water bodies, such as tap water and the Yangtze River, was also investigated. The BPTPA@PAN‐NFM/PMS system demonstrated an effective degradation (>98.0% within 360 min) for BPA in actual water bodies (Figure S44, Supporting Information). The slight decrease in performance may be due to the fact that NOM in the actual water bodies block the active site of catalytic membrane and consume ROS.^[^ ^34^ ^]^ In addition, the presence of inorganic anions (Cl^‐^, HCO_3_^‐^, H_2_PO_4_^‐^) and HA also posed negligible inhibition on the degradation of BPA in the BPTPA@PAN‐NFM/PMS system (Figure S45, Supporting Information). It further indicates the superior performance of the catalytic membrane. Overall, the catalytic performance of as‐prepared BPTPA@PAN‐NFM surpassed that of numerous state‐of‐the‐art PMS‐based AOP systems, with the highest *K* value (>90% of removal efficiency) of the BPTPA/PAN‐NFM membrane system under a durable ultra‐high water flux (Figure 4h).^[^ ^30^, ^34^, ^35^ ^]^

**Figure 5:**
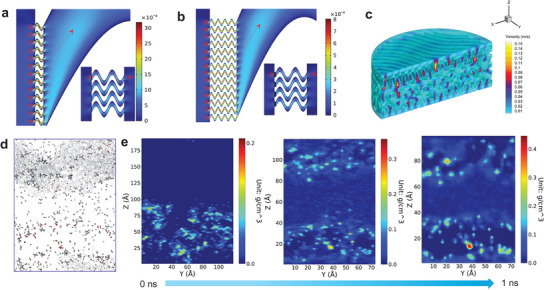
Dynamics simulations. The diffusion velocity of liquid on mesopores among a) sme‐ma‐c and b) lme‐ma‐c (partially enlarged channels), with inset showing the whole channels. c) The diffusion velocity of liquid on macropores among interwoven nanofibers. The color bars in a–c represent the velocity (m s^−1^). d) The distribution of BPA molecules at 1 ns for the diffusion system. e) The densities of BPA molecules at different time for the diffusion system. The color bar in e represents the densities of molecules (g cm^−3^).

In pursuit of a more profound understanding of the influence of BPTPA@PAN‐NFM, characterized by hierarchical pores, on the augmentation of permeability and mass transfer, computational fluid dynamics (CFD) and molecular dynamics (MD) simulations were then carried out. The investigation encompassed four distinct scenarios: two configurations of hierarchically porous membranes, denoted as short and long mesoporous channels integrated with macroporous channels (referred to as sme‐ma‐c and lme‐ma‐c, respectively), single macroporous channels (ma‐c), and single mesoporous channels (me‐c). Diverse sine functions were employed to construct these scenarios (**Figure** **5**a,b and Figure S46 in Supporting Information). The calculated out‐velocities for each scenario were ascertained as follows: ma‐c exhibited a velocity of 3.28 mm s^−1^, sme‐ma‐c showed 0.13 mm s^−1^, lme‐ma‐c registered 0.031 mm s^−1^, and me‐c recorded 0.038 mm s^−1^. It is noteworthy that the thickness of the mesoporous channels within the hierarchical structure exhibited a profound influence on water flux, with thinner mesoporous channels preserving higher velocities within the macroporous channels. Furthermore, adhering to Bernoulli's principle,^[^ ^36^ ^]^ the porous structural design within the fabricated membranes substantially amplified mass transfer rates by facilitating an increase in flow velocity as the flow volume underwent constriction. As exemplified in Figure 5c, we developed a 3D porous model featuring an average pore size of 2 µm, generated through interwoven nanofibers. CFD simulations demonstrated a discernible escalation in flow velocity, transitioning from ≈0.012 to 0.16 m s^−1^ as the fluid traversed the nanofiber matrix, indicating a noteworthy enhancement in mass transfer. In the context of the microporous reactor (Figure 5d and Figure S47 in Supporting Information), MD simulations were subsequently conducted. Analogously, our findings unveiled a concentration effect within the confines of the micropores, manifesting as a substantial increase in the densities of BPA within these restricted regions (from 0.21 to 0.44 g cm^−3^) (Figure 5e). This localized promotion effect is posited to facilitate the acceleration of electron transfer processes, thereby enabling the robust and expeditious degradation of pollutants under conditions of ultrahigh water flux.

## 3 Conclusion

Inspired by the structural functions of lymphatic vessels in immunosurveillance and fluid transport, we have prepared a hierarchical porous catalytic membrane based on nitrogen‐rich CMP (PTPA). The intrinsic microporous nature of PTPA generated an angstrom‐confined system for activating PMS to generate almost 100% ^1^O_2_ for degradation of organic micropollutants, avoiding the self‐degradation of the organic membrane. Experimental investigations and DFT calculations showed that the thermodynamically favorable spontaneous dissociation of PMS occurred in this angstrom‐confined space (with a pore size below ≈7.8 Å), with the cleavage of S‐O bond and the formation of ^1^O_2_. The bioinspired hierarchical structure, i.e., thin layer of mesoporous structure generated between PTPA nanoparticles with micropores merging into macroporous channels between interwoven nanofibers, allows the high mass transfer efficiency and high flux, as CFD and MD simulations’ results indicated. Such a bioinspired catalytic membrane therefore shows a durable high‐efficiency degradation performance (*K* = 1430.9 min^−1^, >95.0% degradation efficiency, 100 h) at a superior permeance (2574 L m^−2^ h^−1^ bar^−1^, 100 h). It is a promising catalytic membrane for the practical degradation of low‐concentration emerging organic micropollutants in wastewater. This study also provides new insights for forming hierarchical pores in catalytic membrane based on porous organic polymer materials for precisely generating ^1^O_2_, enhancing the mass transfer while maintaining superior permeance.

## 4 Experimental Section

### Materials

Tris(4‐bromophenyl) amine (TA), phenylenediamine (PDA), bis(dibenzylideneacetone) palladium(0) (Pd(dba)_2_), sodium tert‐butoxide (NaOtBu), 2‐dicyclohexylphosphino‐2′,4′,6′‐triisopropylbiphenyl (XPhos), NaF and all the solvents with A.R. and C.R. grades were used as received and purchased from Tokyo Chemical Industry (TCI). PAN (*M*_w_ = 150 000), phenol, BPA, HQ, TC, RB5, ARG, RhB, MB, PMS (2KHSO_5_·KHSO_4_·K_2_SO_4_), DMPO and TEMP were purchased from Sinopharm group chemical reagent Co. Ltd, China. All aqueous solutions were prepared with the deionized water from a Master‐S30 HHitech apparatus.

### Synthesis of PTPA

The N‐rich CMP (PTPA) was synthesized by a Buchwald‐Hartwig (BH) cross‐coupling reaction. A Schlenk tube was charged with TA (0.5 mmol), PDA (0.33 mmol), Pd(dba)_2_ (0.03 mmol), XPhos (0.045 mmol), NaOtBu (3.5 mmol), and NaF (0.5 mmol) and was placed under a nitrogen atmosphere. Anhydrous THF (30 mL) was added and the reaction mixture was heated under stirring at 65 °C. After 48 h, the reaction was cooled to room temperature and solvents were then removed by centrifugation. The products were then washed with CHCl_3_, methanol and hot MQ water (12 h each), and then dried in a vacuum. A control experimental was carried out without adding NaF and the obtained sample was labelled as PTPA‐0.

### Membrane Fabrication

The hierarchical porous catalytic membrane was prepared in two steps. In the first step, a PDA@PAN nanofibers mat (PDA@PAN‐NFM) was prepared by electrospinning. 1.5 g of PAN and 1.5 g of PDA were dissolved in 8.5 g of DMF and stirred for 3 h to form a uniformly dispersed PDA/PAN electrospinning solution. The working voltage, the tip‐to‐collector distance, and the flow rate were set at 15 kV, 20 cm, and 1.0 mL h^−1^, respectively. Then the prepared PDA@PAN‐NFM was vacuum dried at room temperature for 12 h. In the second step, BPTPA@PAN‐NFM was prepared by in situ BH cross‐coupling reaction on the PDA@PAN‐NFM. In brief, a Schlenk tube was charged with PDA@PAN‐NFM (100 mg), TA (0.5 mmol), PDA (0.33 mmol), Pd(dba)_2_ (0.03 mmol), XPhos (0.045 mmol), NaOtBu (3.5 mmol) and NaF (0.5 mmol) was placed under a nitrogen atmosphere. Anhydrous THF (30 mL) was added and the reaction mixture was heated under stirring at 65 °C. After 48 h, the BPTPA@PAN‐NFM was obtained after washing and drying. And then PPTPA@PAN‐NFM was prepared by controlling half of the concentration of BH cross‐coupling reaction, that is, PDA@PAN‐NFM (100 mg), TA (0.25 mmol), PDA (0.165 mmol), Pd(dba)_2_ (0.015 mmol), XPhos (0.0225 mmol), NaOtBu (1.75 mmol) and NaF (0.25 mmol). In addition, the PDA@PAN‐NFM was hot pressed to reduce the reactive sites on the surface of the fiber membrane, and then the in situ BH reaction was performed using the same reaction concentration as that used to synthesize the BPTPA@PAN‐NFM to obtain PTPA@PAN‐NFM.^[^ ^37^ ^]^

### Characterization

Fourier transform infrared (FT‐IR) spectral analyses were carried out on a Nicolet 670 spectrometer. X‐ray photoelectron spectra (XPS) were obtained on ESCALAB 250Xi apparatus with C1s signal at 284.4 eV as the reference. The crystallinity of the samples were characterized by a Bruker D8 ADVANCE X‐ray diffraction spectrometer (XRD). Elemental analysis (EA) of samples were tested on a Euro Vector EuroEA3000 instrument. Thermogravimetric analysis (TGA) was measured on a Libra 209F1 instrument under N_2_ atmosphere in the temperature ranging from room temperature to 800 °C, with heating rate keeping at 10 °C min^−1^. Solid‐state ^13^C cross‐polarization magic angle spinning nuclear magnetic resonance (^13^C CP/MAS NMR) spectral analyses were performed on an AVANCE400 spectrometer. The surface areas were calculated from the Brunauer‐Emmett‐Teller (BET) model using the adsorption branches of the N_2_ isotherms in the low‐pressure range from 0.05 to 2.0 at 77 K. Scanning electron microscope (SEM) images were obtained on a Regulus8230 and transmission electron microscope (TEM) images were obtained on a JEM‐2100 TEM microscope. Electron paramagnetic resonance (EPR) spectra were obtained with an EMXnano spectrometer (Bruker, Germany). Zeta potentials of samples were tested on an Anton Paar Litesizer 500 apparatus. UV−vis absorption spectra were achieved using a Shimadzu UV2610 spectrophotometer in the range of 200−800 nm. Total organic carbon (TOC) of treated water was measured on a Elementar Vario TOC instrument.

### Catalytic Performance Evaluation

The catalytic performance of as‐prepared materials was evaluated by activating PMS for BPA degradation in water. Thus, in a typical test, 4 mg catalyst was added into 20 mL of aqueous solution and the suspension was ultrasonically dispersed for 10 min. The initial solution pH was adjusted with 1.0 mol L^−1^ H_2_SO_4_ and 1.0 mol L^−1^ NaOH. Then the catalytic reaction was initiated by adding a certain amount of PMS into the mixture. During the experiment, 1 mL of samples were withdrawn at given time intervals and filtered by a membrane (0.22 µm) to remove solid catalysts for BPA analysis. Three groups of distinct samples were measured for error analysis. The pH of actual solution was maintained using a buffer solution (PBS). The concentration of phenol, hydroquinone (HQ), BPA and TC were analysed by high‐performed liquid chromatography (HPLC, Ultimate 3000; Thermo) with a C18 column at *λ*_DAD_ = 230 nm. The concentration of reactive black 5 (RB5), acid red G (ARG), methylene blue (MB) and rhodamine B (RhB) were measured on a Shimadzu UV2610 spectrophotometer at *λ*_max_. Meanwhile, the mobile phase of phenol, HQ, BPA and TC were a mixture of methanol/MQ water (60:40 v/v), methanol/MQ water (60:40 v/v), methanol/MQ water (70:30 v/v) and acetonitrile/MQ water (65:35 v/v), respectively. The adsorption performance of these pollutants was tested under the same conditions without the addition of PMS. For the catalytic degradation experiments of catalytic membranes, the dead‐end filtration operation was used. The catalytic membranes were tested for membrane flux and BPA degradation rate under a pressure of 0.2 bar. The filtrate was taken at regular intervals and the filtrate was immediately tested for BPA concentration analysis. For the pressure‐dependent continuous‐flow operation, the experiment was carried out for 120 h on a dead‐end unit under a pressure of 0.2 bar.

### DFT Calculations

Three different confined PTPA fragment were obtained from the above simulated PTPA model. The molecular structure of PTPA fragment, PTPA fragment‐PMS and PMS models were optimized using Gaussian 09 software package^[^ ^38^ ^]^ within density functional theory (DFT) at B3LYP/6‐311G (d,p) level. The interaction energies (*E*_int_) was calculated by the following Equation (1) according to Yong:^[^ ^39^ ^]^

(1) $$E_{\mathrm{int}}=E_{\mathrm{PTPA}\;\mathrm{fragment}-\mathrm{PMS}}-E_{\mathrm{PTPA}\;\mathrm{fragment}}-E_{\mathrm{PMS}}$$

where *E*_PMS_ is the total energy of PMS, *E*_PTPA fragment_ is the total energy of PTPA fragment and *E*_PTPA fragment‐PMS_ is the total energy of the new system formed between the PTPA fragment and PMS. Total energy is the energy of optimized molecular structure calculated using Gaussian 09 software package within DFT at B3LYP/6‐311G (d, p) level in the separate system or formed new system.

The transition states were searched by the climbing nudged elastic band (CINEB) method using CP2K software.^[^ ^35f^ ^]^ The cluster was placed at the center of a 23.5 × 23.5 × 23.5 Å supercell with a sufficient thickness of vacuum layer, allowing for calculations to be performed as a 0D system. The semiempirical GFN1‐xTB^[^ ^40^ ^]^ method was applied to describe the ion‐electron exchange‐correlation, in conjunction with Grimme's D3 dispersion corrections.^[^ ^41^ ^]^

The Multiwfn package is used for wave‐function analyses of EDD.^[^ ^42^ ^]^ The VMD software is used for visualization.^[^ ^43^ ^]^

The independent gradient model based on Hirshfeld partition of molecular density (IGMH) was adopted in the visual analysis of intermolecular interaction of the optimized PTPA and PMS, which was performed on Multiwfn software. Weak interaction regions were visualized with a Sign(λ2)ρ colored isosurfaces isovalue of 0.005 a.u.^[^ ^44^ ^]^

### CFD Simulation

Four specimens were established with COMSOL.^[^ ^45^ ^]^ In the first case, the channels have a width of 2 µm and consist of four periodic sine functions, while the porosity remains constant at 67%. In the second configuration, adjacent to the micron‐sized channel comprised of two periodic sine functions with a width of 2 µm, there are 20 mesoporous channels, each governed by a single periodic sine function with a width of 50 nm, merging into the micron‐sized channel. The porosity for this setup is fixed at 56%. In the third case, similar to the second, there are 20 mesoporous channels in front of the micron‐sized channel with two periodic sine functions of 2 µm, but in this case, these mesoporous channels are characterized by four periodic sine functions with a width of 50 nm, again merging into the micron‐sized channel, with a porosity of 56%. The final configuration consists of a mesoporous channel with a diameter of 50 nm, featuring four periodic sine functions and a fixed porosity of 20%. To simulate the fluid dynamics within the fibrous materials, a 3D porous model is formed by the intersection of 10 layers of nanofibers, with an average pore size of 2 µm. In the initial calculation, the fluid (water) height within the cylinder is set at 2 m, and the cross‐sectional area measures 3.14 × 10^‐4^ m^2^. The fluid flow is assumed to be a laminar flow of an incompressible Newtonian fluid characterized by a density (*ρ*) of 1000 kg m^−3^ and a viscosity (*µ*) of 2.98 × 10^−3^ Pa s.

### MD Simulation

The GROMACS software was used to performed the MD simulations.^[^ ^46^ ^]^ The permeation of BPA through the microporous reactor constructed above was predicted via a typical simulation system illustrated in Figure 5D. The PTPA model was placed in the middle of the simulation box, and a periodic‐boundary condition was applied in the *x*−*y* direction. The BPA molecules were placed on the left of the PTPA model along the *z* direction, and were fixed on the *x*−*y* direction while being movable along the *z* direction. For the dynamic process, the annealing was first conducted. The temperature was gradually increased form 0 to 300 K within 200 ps, then the simulation system was relaxed by a 1 ns constant‐temperature, constant‐pressure (NPT) simulation at 300 K to achieve pressure equilibrium. Subsequently, the 1 ns NPT dynamics were simulated at 298 K and 0.2 bar. The trajectory of the last 100 ps was collected for data processing and analysis.

## Conflict of Interest

The authors declare no conflict of interest.

## Supporting information

Supporting Information

## Data Availability

The data that support the findings of this study are available from the corresponding author upon reasonable request.
