# Supplementary material for: Conjugated Microporous Polymers‐Based Catalytic Membranes with Hierarchical Channels for High‐Throughput Removal of Micropollutants
Source: Adv Sci (Weinh). 2024 Jun 3;11(29):2401966. doi: 10.1002/advs.202401966 (PMC11304305; doi:10.1002/advs.202401966)
Supplement: Supplementary file 1 — Supporting Information [file ADVS-11-2401966-s001.docx]

Supporting Information

Conjugated Microporous Polymers-based Catalytic Membranes with Hierarchical Channels for High-Throughput Removal of Micropollutants

Jiaqiang Li, Wei Lyu^*^, Xuejin Mi, Cheng Qian, Yanbiao Liu, Junrong Yu, Richard B. Kaner, Yaozu Liao^*^

Contents

**Supplementary Text** 4

**1**. Synthesis of PANI 4

**2**. Decomposition efficiency of PMS 4

**3**. Electrochemical measurement 4

**4**. Calculation of retention time 5

**5**. Calculation of reaction rate constant 5

**6**. Kinetic modeling 6

**7**. Computational methodology 6

**Supplementary Figures** 7

**Scheme S1**. The synthetic route and chemical structure of PTPA. 7

**Figure S1**. ^13^C CP/MAS NMR spectra of PTPA. 8

**Figure S2**. C 1s XPS spectra of PTPA. 8

**Figure S3**. TGA scans of PTPA. 9

**Figure S4**. (a) SEM, (b) TEM, and (c) HRTEM images of PTPA. 9

**Figure S5**. Removal efficiency of BPA. BPA was adsorbed on PTPA in the first 30 min and then degraded by the addition of PMS. 10

**Figure S6**. The ﬁrst-order kinetics ﬁtting of BPA catalytic degradation under different reaction systems. 10

**Figure S7**. The decomposion efficiency of PMS in different systems. 11

**Figure S8**. Removal efficiency of BPA in different PTPA/PMS system. 11

**Figure S9**. Inﬂuences of (a) Cl^−^, (b) H_2_PO_4_^−^, (c) HCO_3_^−^ and (d) HA on BPA degradation. 12

**Figure S10**. pH variation during the reaction in the PTPA/PMS system. 12

**Figure S11**. (a) Inﬂuences of different pHs (maintained with a buffer solution) on BPA removal efficiency. (b) The first-order rate constant of the corresponding conditions. 13

**Figure S12**. pH variation during the reaction in the PTPA/PMS system containing HCO_3_^-^. 13

**Figure S13**. Comparison of BPA removal efficiency of PTPA/PMS system containing EtOH, Cl^—^, and a mixture of Cl^—^ and EtOH. 14

**Figure S14**. Inﬂuences of (a) initial BPA concentration, (b) different catalyst dose, (c) PMS concertation and (d) reaction temperature on BPA removal efficiency. 14

**Figure S15**. The first-order rate constant under (a) initial BPA concentration, (b) different catalyst dose, (c) PMS concertation and (d) reaction temperature on BPA removal efficiency. 15

**Figure S16**. Eﬀects of FFA and β-carotene on BPA degradation of PTPA/PMS system. 16

**Figure S17**. EPR spectra of different systems using DMPO as trapping agent in the PTPA/PMS system. 16

**Figure S18**. SEM images of PTPA before (a-b) and after (c-d) use. (e) FT-IR spectra of PTPA before and after use. 17

**Figure S19**. EPR spectra of different systems using TEMP as trapping agent in the PANI/PMS system. 17

**Figure S20**. Comparison of BPA removal efficiency (a) and first-order rate constant under different radical scavengers (b) of PANI/PMS system. 18

**Figure S21**. N 1s XPS spectra of PANI. 18

**Figure S22**. (a) N_2_ adsorption-desorption isotherms (77 K) and (b) pore size distribution of PTPA-0; (c) removal efficiency of BPA on PTPA-0 and PTPA-0/PMS system and (d) the first-order rate constant under different radical scavengers of PTPA-0/PMS system. 19

**Figure S23**. (a) N_2_ adsorption-desorption isotherms (77 K) and (b) pore sized distribution of PANI-C. (c) Removal efficiency of BPA on PANI-C and PANI-C/PMS system. 19

**Figure S24**. BPA removal efficiency of PTPA/PMS system under different atmosphere: nitrogen gas, air, and oxygen gas. 20

**Figure S25**. EPR spectra of PTPA/PMS system using TEMP as trapping agent with or without addition of p-BQ. 20

**Figure S26**. Optimized geometric structure of PTPA-oligomer. 21

**Figure S27**. (a) Pore architecture in the structural model of PTPA. Purple region indicates the void space for N_2_ and (b) the calculated pore size distribution of PTPA. 21

**Figure S28**. Three different confined fragmentation with pore size with van der Waals diameter of 7.5, 4.9 and 3.3 Å, respectively. 22

**Figure S29**. Optimized PMS structure using B3LYP/CC-pCTZ basic set. 22

**Figure S30**. Energetic and geometric data of PTPA and PMS under different pore sizes. 23

**Figure S31**. Zeta potential values of PTPA. 23

**Figure S32**. LSV curves of PTPA. 24

**Figure S33**. BPA degradation intermediates ([P+Na]^+^) in the PTPA/PMS system. 25

**Figure S34**. Degradation pathways of BPA degradation by catalytic PMS using PTPA. 26

**Figure S35**. Adsorption efficiency of PTPA for organic pollutants with different molecular sizes. 27

**Figure S36**. Removal efficiency of organic pollutants with different sizes by PTPA/PMS system. (a) Removal efficiency of organic pollutants with different size under PTPA/PMS system; (b) correlations of *K_obs_* and molecular size; (c) correlations of *K_obs_* and adsorption capacity; (d) and (e) adsorption efficiency of organic pollutants with different size; (f) mean-squared displacement (MSD) of various pollutants through PTPA. 27

**Figure S37**. Simulation snapshots of (a) phenol, (b) HQ, (c) BPA and (d) TC molecules diffusing into the PTPA pore voids at 0 ps, 10 ps, 100 ps and 1000 ps. 28

**Figure S38**. SEM images of (a) PAN-NFM, (b) PTPA@PAN-NFM, (c) PPTPA@PAN-NFM and (d) BPTPA@PAN-NFM. 29

**Figure S39**. The photographs of BPTPA@PAN-NFM with different sizes. 29

**Figure S40**. (a) FT-IR spectra and (b) XRD patterns of BPTPA@PAN-NFM before and after continuous-flow operation (120 h). 30

**Figure S41**. SEM images of BPTPA@PAN-NFM before (a-b) and after (c-d) continuous-flow operation (120 h). 30

**Figure S42**. The photographs and microscope images of the support membrane before (a-b) and after (c-d) continuous-flow operation (120 h). 31

**Figure S43**. Removal efficiency of different pollutants in the BPTPA@PAN-NFM/PMS system. 31

**Figure S44**. Influence of tap water and the Yangtze River on pollutant removal in the BPTPA@PAN-NFM/PMS system. 32

**Figure S45**. The effect of different anions, HA, and coexisted ions and HA on the BPA removal efficiency in the BPTPA@PAN-NFM/PMS system. 32

**Figure S46**. The diffusion velocity of liquid on mesopores among (a) ma-c and (b) me-c. 33

**Figure S47**. The distribution of BPA molecules at 0 ns for the diffusion system. 33

**Supplementary Tables** 33

**Table S1**. Elemental analysis of PTPA and PANI. 33

**Table S2**. Parameters of pore structures of samples by N_2_ adsorption isotherms. 34

**Table S3**. Comparison between PTPA and the recently reported metal-free catalysts in the catalytic degradation activity on BPA. 34

**Table S4**. The effect of different anions, HA, and coexisted ions and HA on BPA degradation in the PTPA/PMS system. 35

**Table S5**. Kinetic parameters of adsorption of organic pollutants with different molecular sizes on PTPA. 36

**Table S6**. The structural characteristics and removal performance of PTPA@PAN-NFM, PPTPA@PAN-NFM and BPTPA@PAN-NFM membranes. 36

**References** 37

# Supplementary Text

# 1. Synthesis of PANI

PANI was prepared by a common oxidation polymerization of aniline monomer. In brief, 1 mL of monomer aniline was dissolved in 30 mL of H_2_SO_4_ water solution (0.5 M). 20 mL of H_2_SO_4_ water solution (0.5 M) containing the FeCl_3_ was then added dropwise, and the resulting mixture was stirred at room temperature for 24 h. Finally, the product was collected by vacuum filtration, washed with distilled water and ethanol several times, and then dried in a vacuum furnace at 60 °C for 24 h. A control experimental was carried out with adding NaF (0.5 mmol) and the obtained sample was labelled as PANI-C.

# 2. Decomposition efficiency of PMS

The decomposition efficiency of PMS was determined as follows.^[1]^ In brief, 1 mL of the reacted solution collected at different time was added into a 10 mL glass tube containing 1 mL solution (KI (0.5 g L^−1^) and NaHCO_3_ (10 g L^−1^)), and the solution was then diluted with deionized water to 10 mL. The resulting solutions were hand shaken and allowed to equilibrate for 15 min. Then PMS concentration was analyzed by a UV–Vis spectrophotometer at 352 nm.

# 3. Electrochemical measurement

The electrochemical measurements were performed in a Gamry Interface 1100E electrochemical workstation at room temperature. The slurry prepared by mixing PTPA (80 wt%), acetylene (10 wt%) and polytetrafluoroethylene (10 wt%) was coated onto a Kuraray carbon paper and then dried at 100 °C overnight in a vacuum oven, affording flexible working electrodes. A three-electrode system was used to measure the individual electrode with Pt wire as the counter electrode and Ag/AgCl electrode as the reference electrode, respectively. All potentials reported in our work were referenced to a reversible hydrogen electrode (RHE). The linear sweep voltammetry (LSV) experiments were measured between 0 and 0.8 V vs RHE at a scan rate of 5 mV s^-1^. All the electrochemical tests were performed at room temperature.

# 4. Calculation of retention time

The calculation method of the solution retention time inside PTPA/PAN membrane is as follows:

$t=\frac{V_{pore}}{Q}=\frac{V_{active}\varepsilon}{Q}=\frac{h_{active}A_{active}\varepsilon}{vA_{active}}=\frac{h_{active}\varepsilon}{v}$ (S1)

where *V_active_*, *A_active_*, *ε*, and *h_active_* are the volume (m^3^), projected area (m^2^), porosity, and the thickness of the catalytic membrane (m), respectively. *Q* is the feed solution flux (L h^-1^), and *v* is the superficial velocity (L m^-2^ h^-1^). The hactive was measured by the SEM image (the cross section) of PTPA/PAN membrane.

# 5. Calculation of reaction rate constant

The reaction rate of the catalytic degradation was measured according to the following pseudo-first-order kinetics equation:

$-ln\frac{C}{C_{0}}=kt$ (S2)

where *C* and *C_0_* are the concentration of initial feed and filtrate (mg L^-1^), *K* (min^-1^) is the reaction rate constant, *t* (min) is the reaction time.

# 6. Kinetic modeling

Two widely used kinetic models, *i.e.*, pseudo-first-order and pseudo-second-order were used to evaluate the adsorption equilibrium of pollutants onto the catalysts, as described by the following, respectively:

$Q_{t}=Q_{e}(1-e^{-K_{1}t})$ (S3)

$Q_{t}=\frac{K_{2}Q_{e}^{2}t}{1+K_{2}Q_{e}t}$ (S4)

where *Q_e_* (mg g^-1^) is the adsorption capacity for the dye at equilibrium, *K_1_* (min^-1^) and *K_2_* (g mg^-1^ min^-1^) are the rate constant for the pseudo-first-order and pseudo-second-order, respectively.

# 7. Computational methodology

**7.1 Generation of structural model for PTPA**

Like the modelling strategy developed by Maji et al., a hypothetical PTPA oligomer was modelled by fusing three PTPA-monomers and optimized first by DFT (B3LYP/6-311G) in Gaussian 09 software.^[2]^ The molecular dynamics (MD) simulations were then used to generate the initial configuration of 30 PTPA-oligomers (total of 7680 atoms) in Materials Studio software package 6.0, using the consistent valence forcefield (CVFF) force field. The subsequent MD simulations for the energy minimization of the initial configuration was performed in LAMMPS package.^[3]^ The details of MD stimulations were followed the reference 4.^[4]^

**7.2 Calculation of pore size distribution of PTPA model**

The established PTPA model was used to analyse the pore size distribution using PoreBlazer v4.0 (PB v4.0) developed by Lev Sarkisov and co-worker.^[5]^ The PB v4.0 was taken as input four files. All the simulations presented in this article have been carried out using the default values.

**7.3 Simulation of pollutant’s diffusion into PTPA model**

The diffusion of four organic contaminants (phenol, HQ, BPA and TC) of different sizes through PTPA constructed above was predicted by a typical simulation system. The three-dimensional box was 33.11×33.91×28.44 Å with a certain number of contaminant molecules (at a density of 1.0 g cm^-3^), which was described with COMPASSII. The system contains the contaminant molecules on the left and the PTPA model on the right. Subsequently, a 1000 ps molecular dynamics simulation at 298 K was performed in the constant-temperature, constant-volume (NVT) ensemble with the COMPASS force ﬁeld. The PTPA kept fixed in the whole simulation process.^[6]^

# Supplementary Figures

Scheme S1. The synthetic route and chemical structure of PTPA.

**
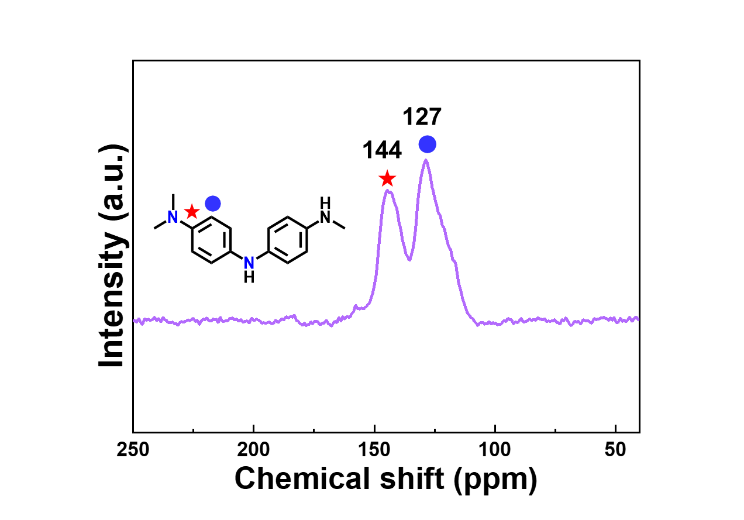
**

Figure S1. ^13^C CP/MAS NMR spectra of PTPA.


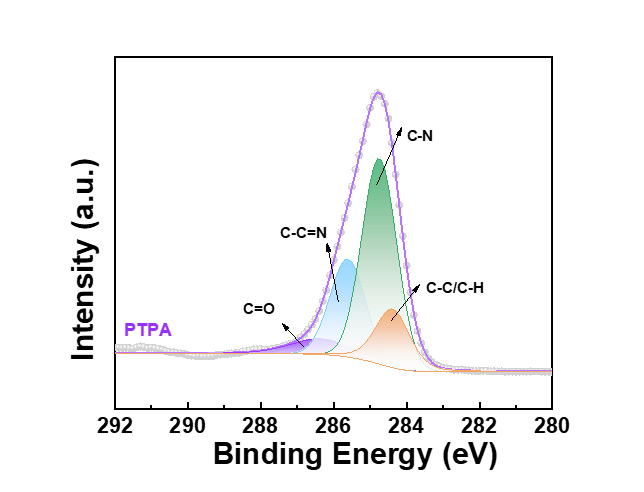


Figure S2. C 1s XPS spectra of PTPA.


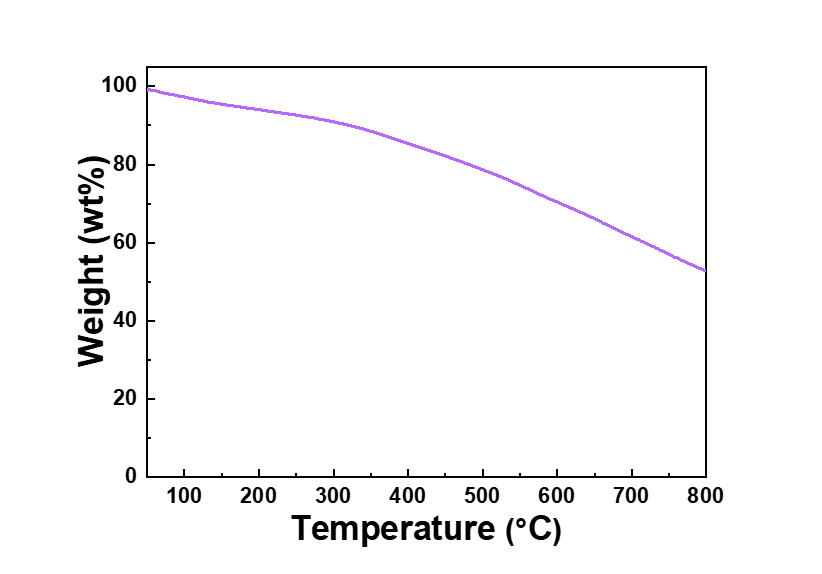


Figure S3. TGA scans of PTPA.


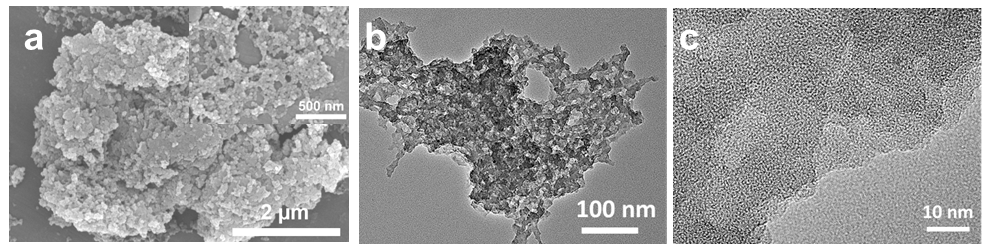


Figure S4. (a) SEM, (b) TEM, and (c) HRTEM images of PTPA.


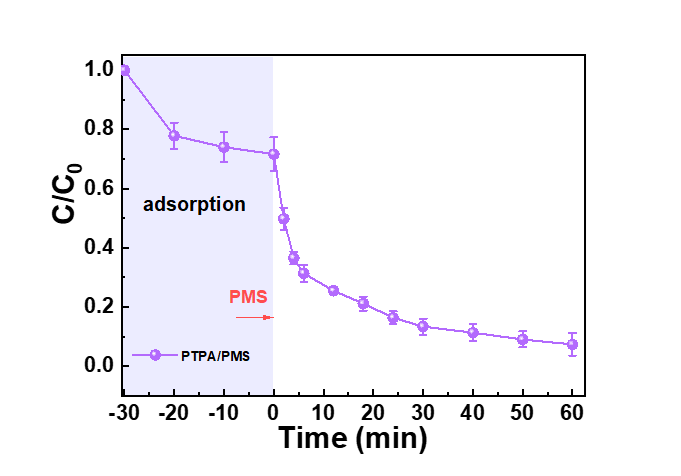


Figure S5. Removal efficiency of BPA. BPA was adsorbed on PTPA in the first 30 min and then degraded by the addition of PMS.

To identify the adsorption contribution, a control experiment, in which BPA was degraded by PMS after adsorption for 30 min, was carried out. Similarly, 86.2% removal efficiency was obtained (**Figure S5**). Notably, an ~7.2 % reduced total organic carbon (TOC) value obtained in 30 min verified that the contribution of adsorption on these removed BPA is less than 7.2%. The obtained results suggested that the adsorbed BPA can be decomposed in the degradation process, otherwise the occupied active sites by the adsorbed BPA would affect the subsequent removal efficiency and the reduced TOC value should be more than 30%. Besides, such a low mineralization suggested the probably existence of ^1^O_2_, which exhibits a moderate nature and can hardly mineralize organic pollutants.


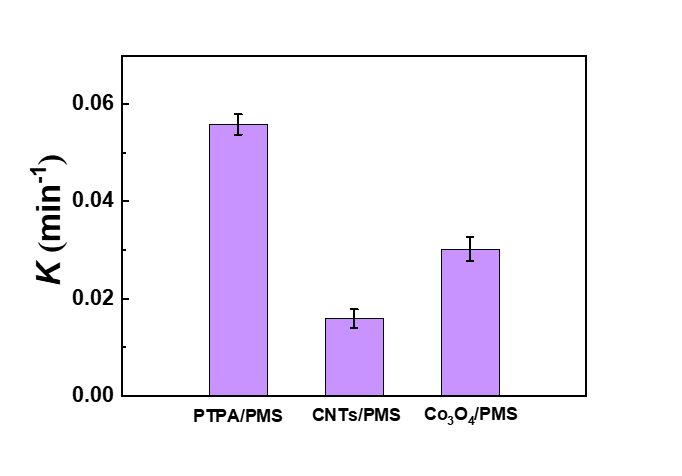


Figure S6. The ﬁrst-order kinetics ﬁtting of BPA catalytic degradation under different reaction systems.

**
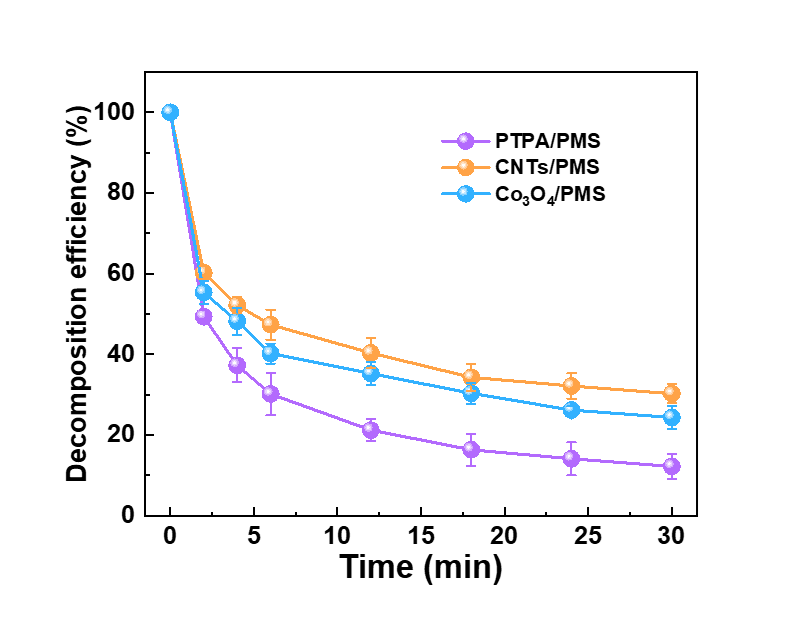
**

Figure S7. The decomposion efficiency of PMS in different systems.


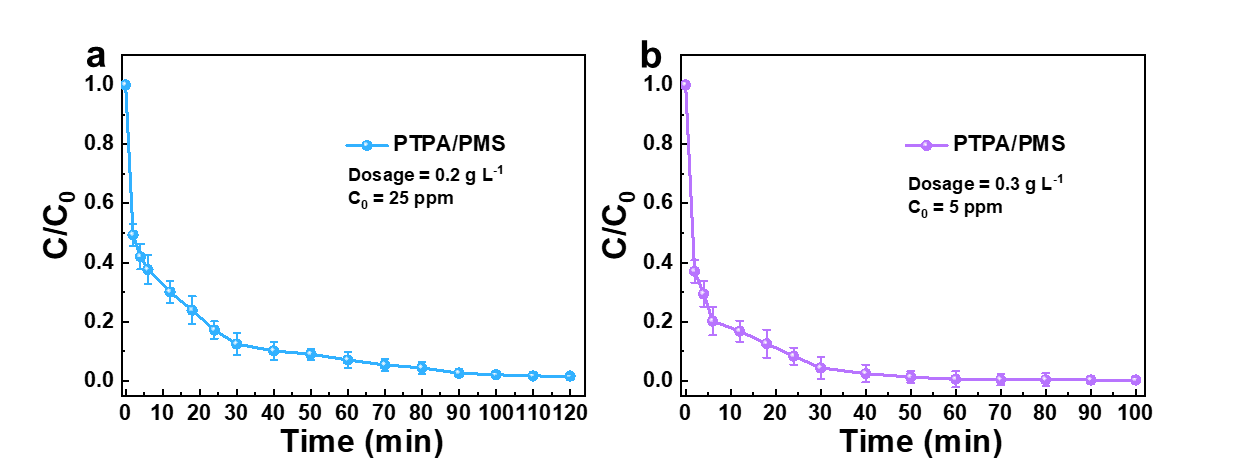


Figure S8. Removal efficiency of BPA in different PTPA/PMS system.


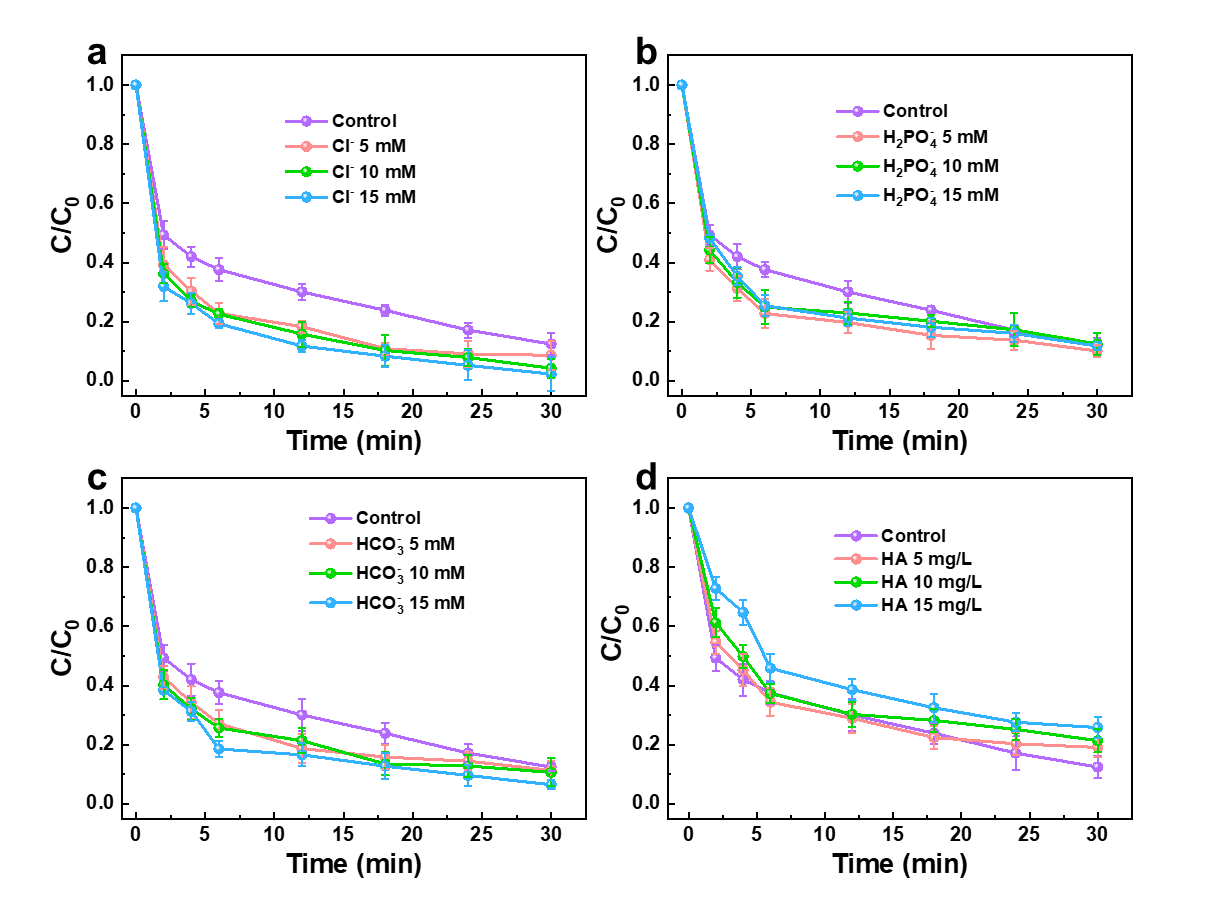


Figure S9. Inﬂuences of (a) Cl^−^, (b) H_2_PO_4_^−^, (c) HCO_3_^−^ and (d) HA on BPA degradation. (Conditions: [Catalyst] = 0.2 g L^-1^, [BPA] = 25 mg L^-1^, [PMS] = 1.0 mmol L^-1^, T = 25 °C, initial pH 6.0)

**
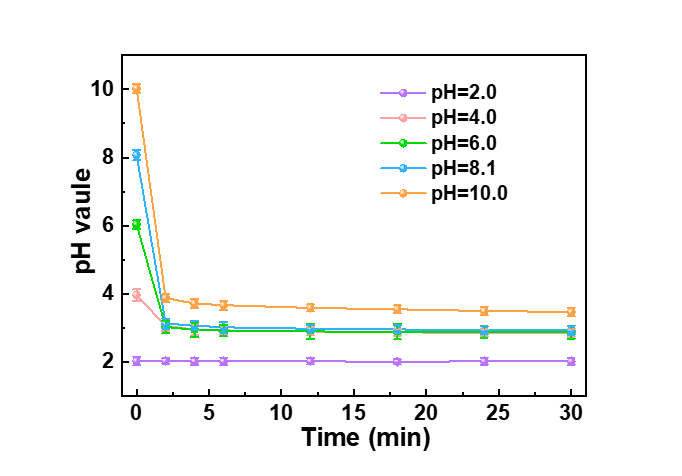
**

Figure S10. pH variation during the reaction in the PTPA/PMS system.


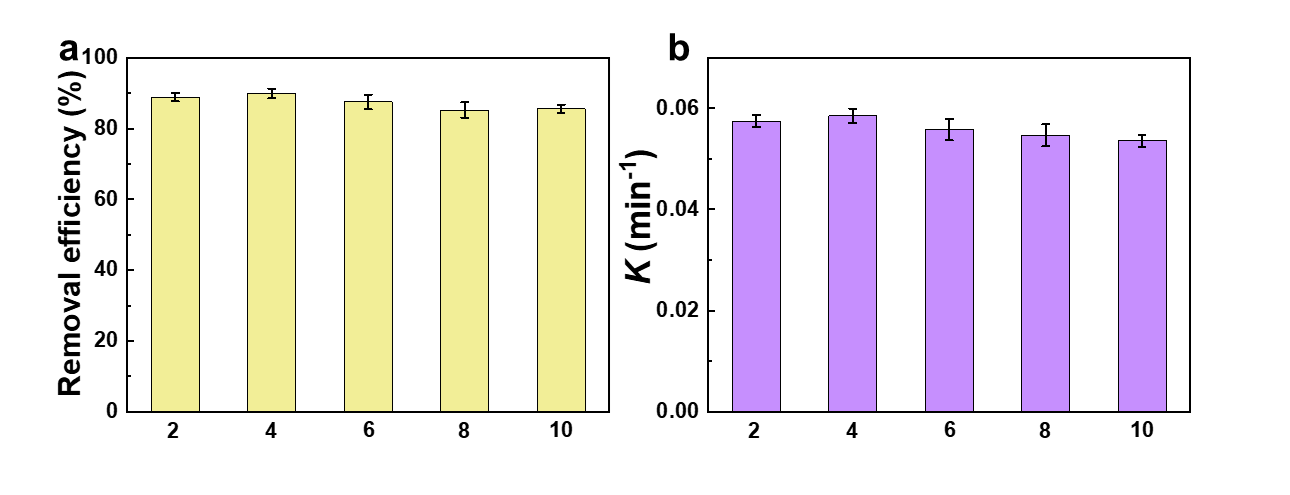


Figure S11. (a) Inﬂuences of different pHs (maintained with a buffer solution) on BPA removal efficiency. (b) The first-order rate constant of the corresponding conditions. (Conditions: [Catalyst] = 0.20 g L^-1^, [BPA] = 25 mg L^-1^, [PMS] = 1.0 mmol L^-1^, T = 25 °C)


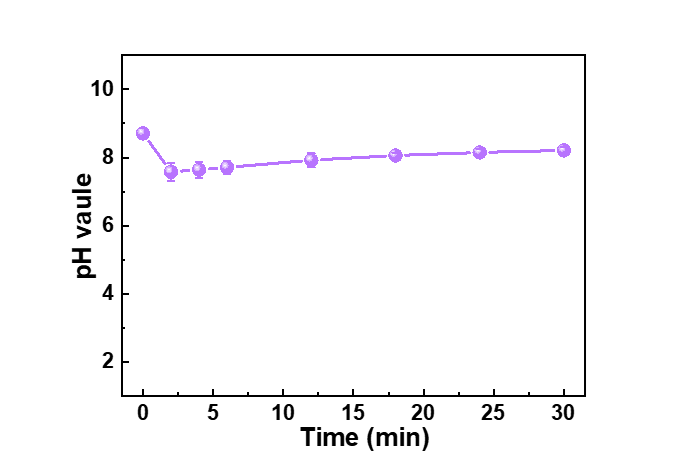


Figure S12. pH variation during the reaction in the PTPA/PMS system containing HCO_3_^-^.


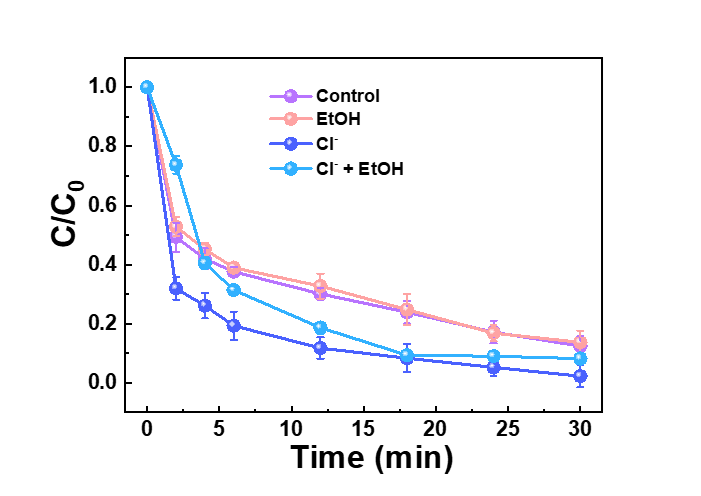


Figure S13. Comparison of BPA removal efficiency of PTPA/PMS system containing EtOH, Cl^—^, and a mixture of Cl^—^ and EtOH. (Conditions: [Catalyst] = 0.2 g L^-1^, [BPA] = 25 mg L^-1^, [PMS] = 1.0 mmol L^-1^, [Cl^—^] = 15 mmol L^-1^, [EtOH] = 2.0 mol L^-1^, T = 25 °C, initial pH 6.0)

**
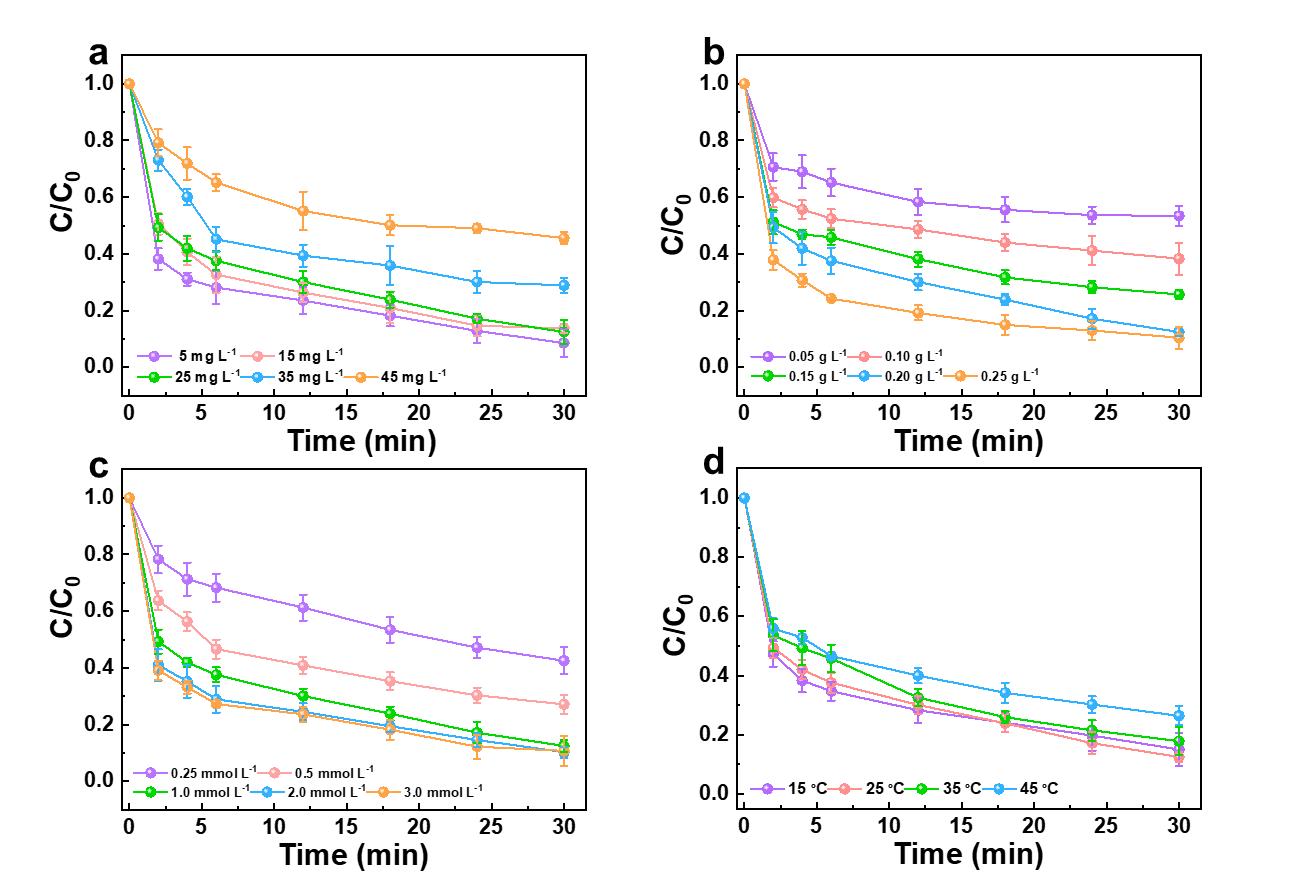
**

Figure S14. Inﬂuences of (a) initial BPA concentration, (b) different catalyst dose, (c) PMS concertation and (d) reaction temperature on BPA removal efficiency. (Conditions: [Catalyst] = 0.20 g L^-1^, [BPA] = 25 mg L^-1^, [PMS] = 1.0 mmol L^-1^, T = 25 °C, initial pH 6.0)

We then systemically evaluated the impact of the initial BPA concentration, PTPA dosage, PMS concentration and reaction temperature on the degradation of BPA (**Figure S14** and **S15**). An acceptable removal performance (>60%) of BPA in the range from 5 to 35 mg L^-1^ was gained (**Figure S14a**). The catalytic performance became worse with increasing BPA concentration. It may be due to the quantitative ROS generated, which resulted in the decreased kinetic constant. When the PTPA dosage was increased from 0.05 to 0.25 g L^-1^, the catalytic performance was remarkably improved owing to the increased active sites (**Figure S14b**). It should be noted that the removal performance was not affected by increasing the PMS concentration from 1.0 to 3.0 mmol L^-1^ (**Figure S14c**), indicating the possibility of a non-radical mechanism in the PTPA/PMS system.^[7]^ **Figure S14d** shows that the reaction rate decreased at higher temperature (45 °C), suggesting the exothermic nature of PTPA/PMS process.

**
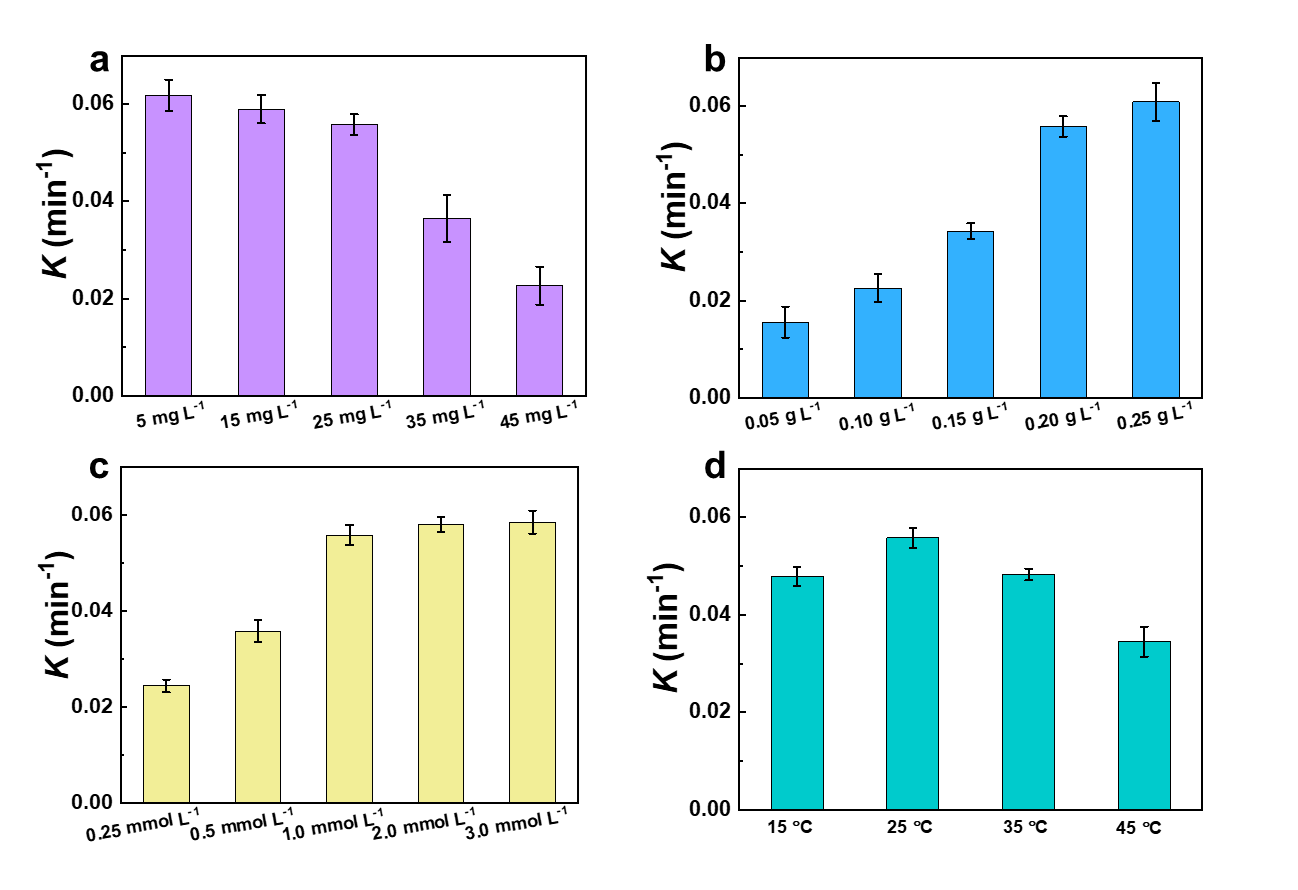
**

Figure S15. The first-order rate constant under (a) initial BPA concentration, (b) different catalyst dose, (c) PMS concertation and (d) reaction temperature on BPA removal efficiency.

**
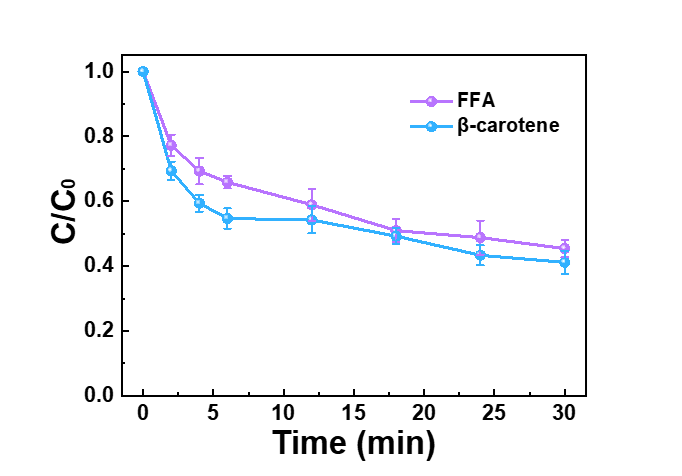
**

Figure S16. Eﬀects of FFA and β-carotene on BPA degradation of PTPA/PMS system. (Conditions: [Catalyst] = 0.2 g L^-1^, [BPA] = 25 mg L^-1^, [PMS] = 1.0 mmol L^-1^, [FFA] = [β-carotene] = 100 mmol L^-1^, T = 25 °C, initial pH 6.0)

**
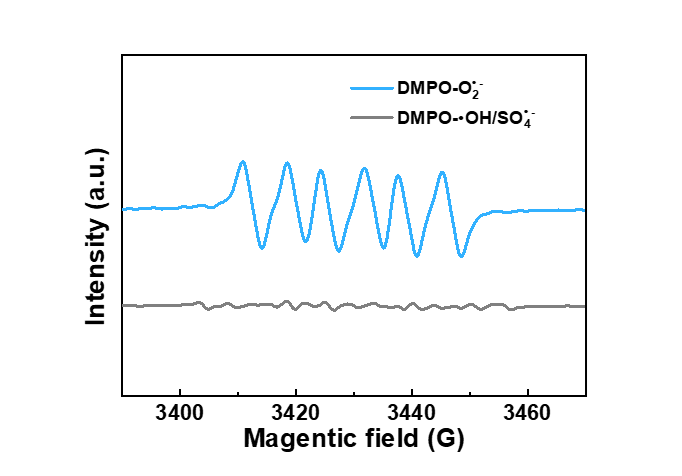
**

Figure S17. EPR spectra of different systems using DMPO as trapping agent in the PTPA/PMS system.


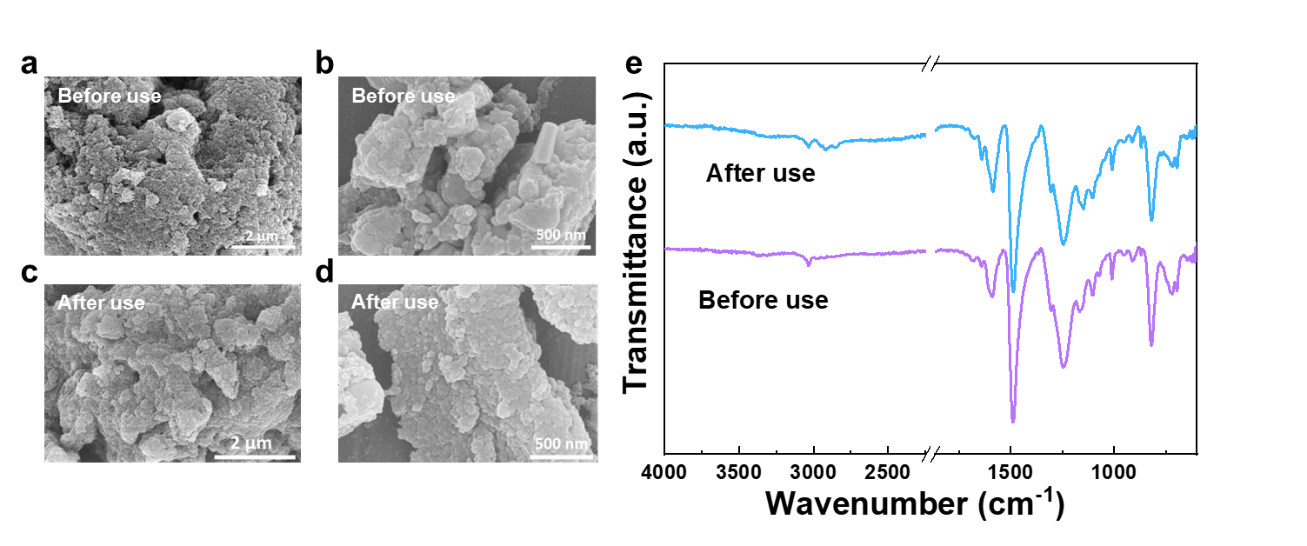


Figure S18. SEM images of PTPA before (a-b) and after (c-d) use. (e) FT-IR spectra of PTPA before and after use.

It can be seen that no obvious change in the chemical structure, composition and morphology observed for PTPA after 5 uses suggested its excellent stability (**Figure S18**).

**
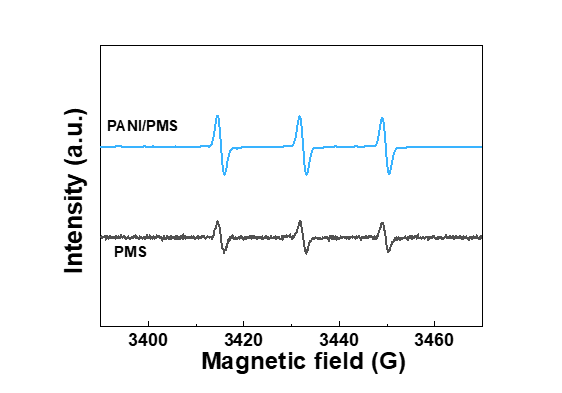
**

Figure S19. EPR spectra of different systems using TEMP as trapping agent in the PANI/PMS system.

**
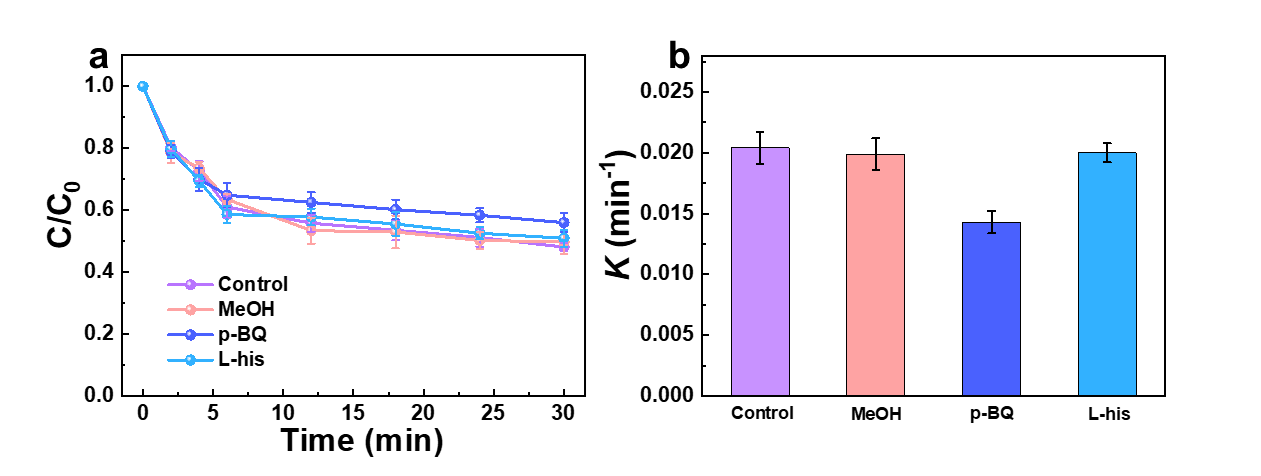
**

Figure S20. Comparison of BPA removal efficiency (a) and first-order rate constant under different radical scavengers (b) of PANI/PMS system. (Conditions: [Catalyst] = 0.2 g L^-1^, [BPA] = 25 mg L^-1^, [PMS] = 1.0 mmol L^-1^, T = 25 °C, initial pH 6.0)

**
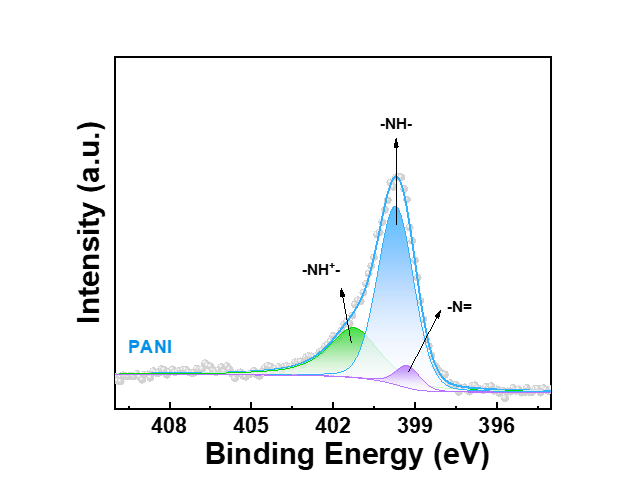
**

Figure S21. N 1s XPS spectra of PANI.

**
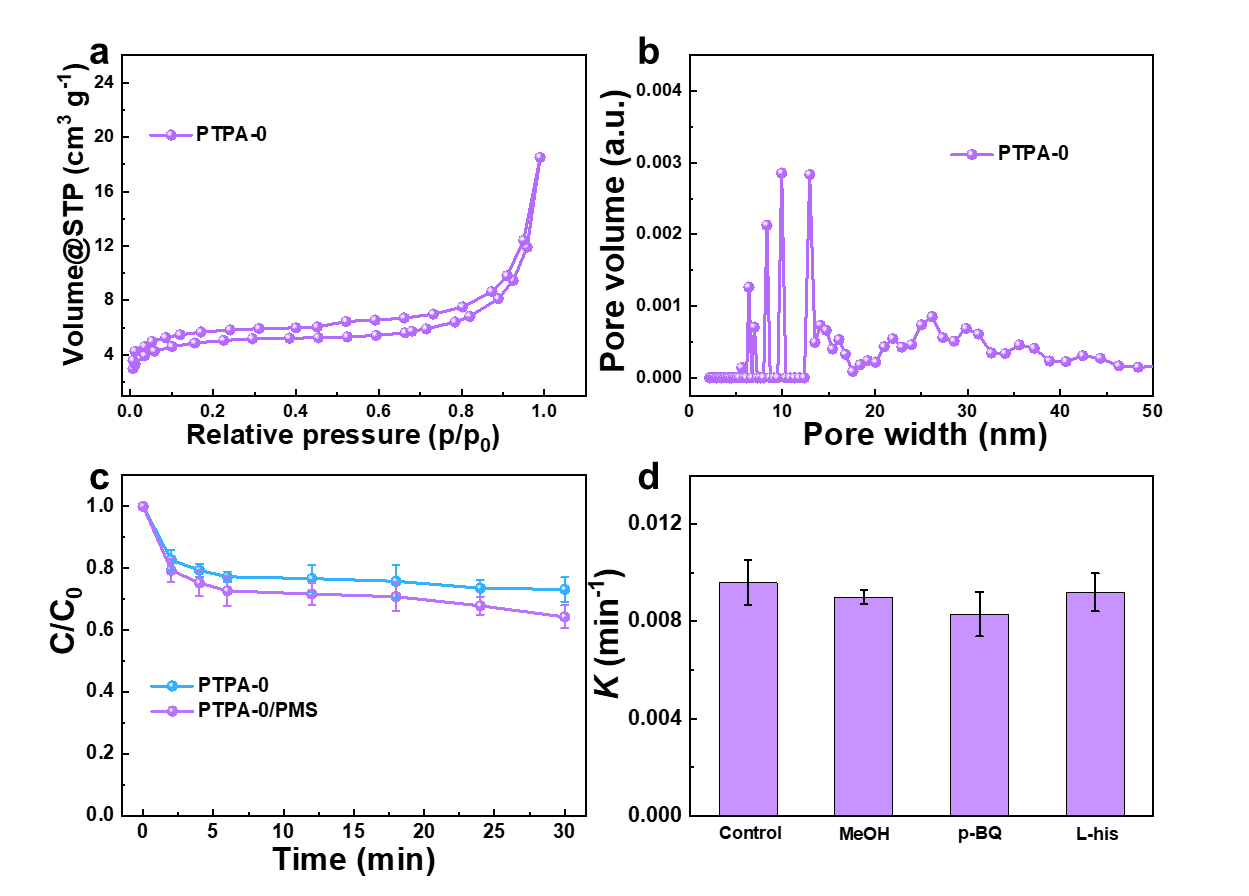
**

Figure S22. (a) N_2_ adsorption-desorption isotherms (77 K) and (b) pore size distribution of PTPA-0; (c) removal efficiency of BPA on PTPA-0 and PTPA-0/PMS system and (d) the first-order rate constant under different radical scavengers of PTPA-0/PMS system.

**
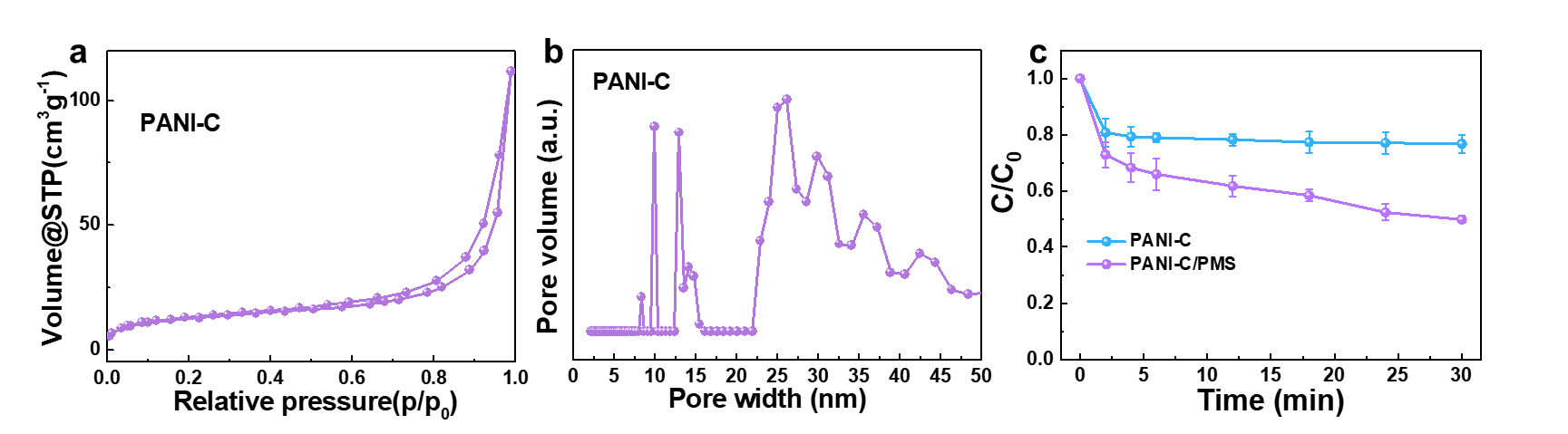
**

Figure S23. (a) N_2_ adsorption-desorption isotherms (77 K) and (b) pore sized distribution of PANI-C. (c) Removal efficiency of BPA on PANI-C and PANI-C/PMS system.

**
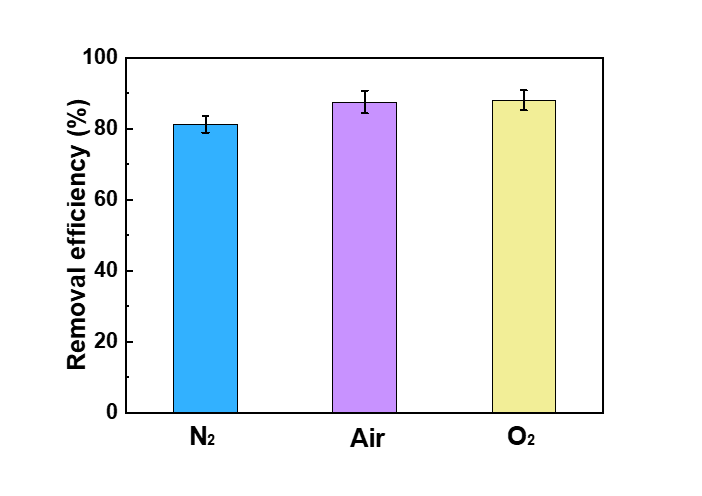
**

Figure S24. BPA removal efficiency of PTPA/PMS system under different atmosphere: nitrogen gas, air, and oxygen gas. (Conditions: [Catalyst] = 0.2 g L^-1^, [BPA] = 25 mg L^-1^, [PMS] = 1.0 mmol L^-1^, T = 25 °C, initial pH 6.0)

**
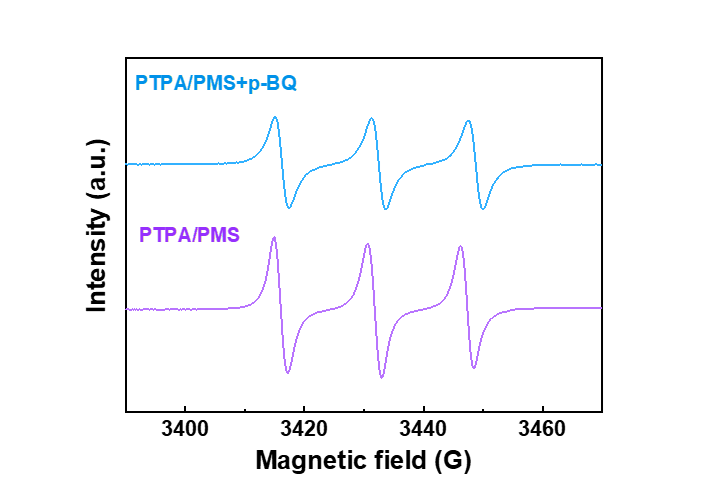
**

Figure S25. EPR spectra of PTPA/PMS system using TEMP as trapping agent with or without addition of p-BQ.


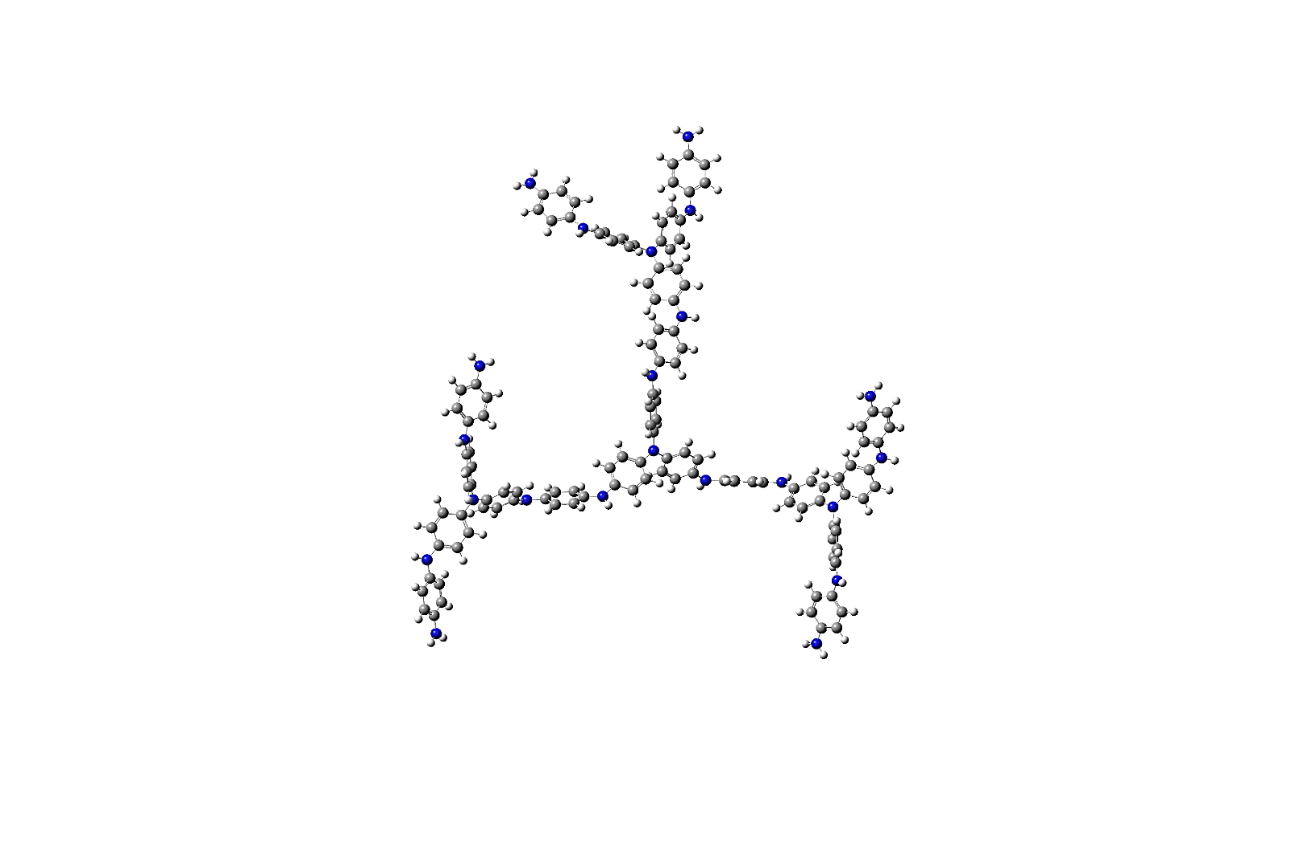


Figure S26. Optimized geometric structure of PTPA-oligomer.


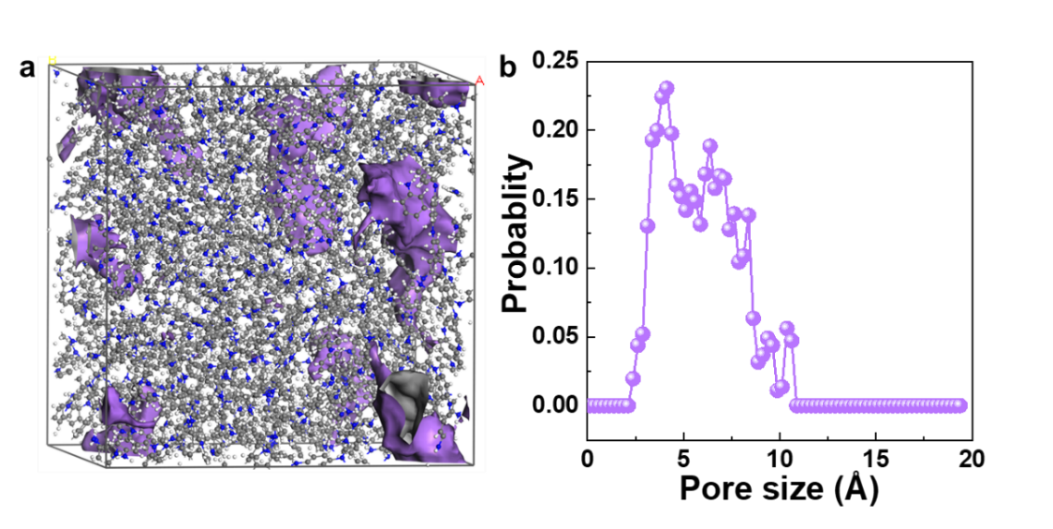


Figure S27. (a) Pore architecture in the structural model of PTPA. Purple region indicates the void space for N_2_ and (b) the calculated pore size distribution of PTPA.


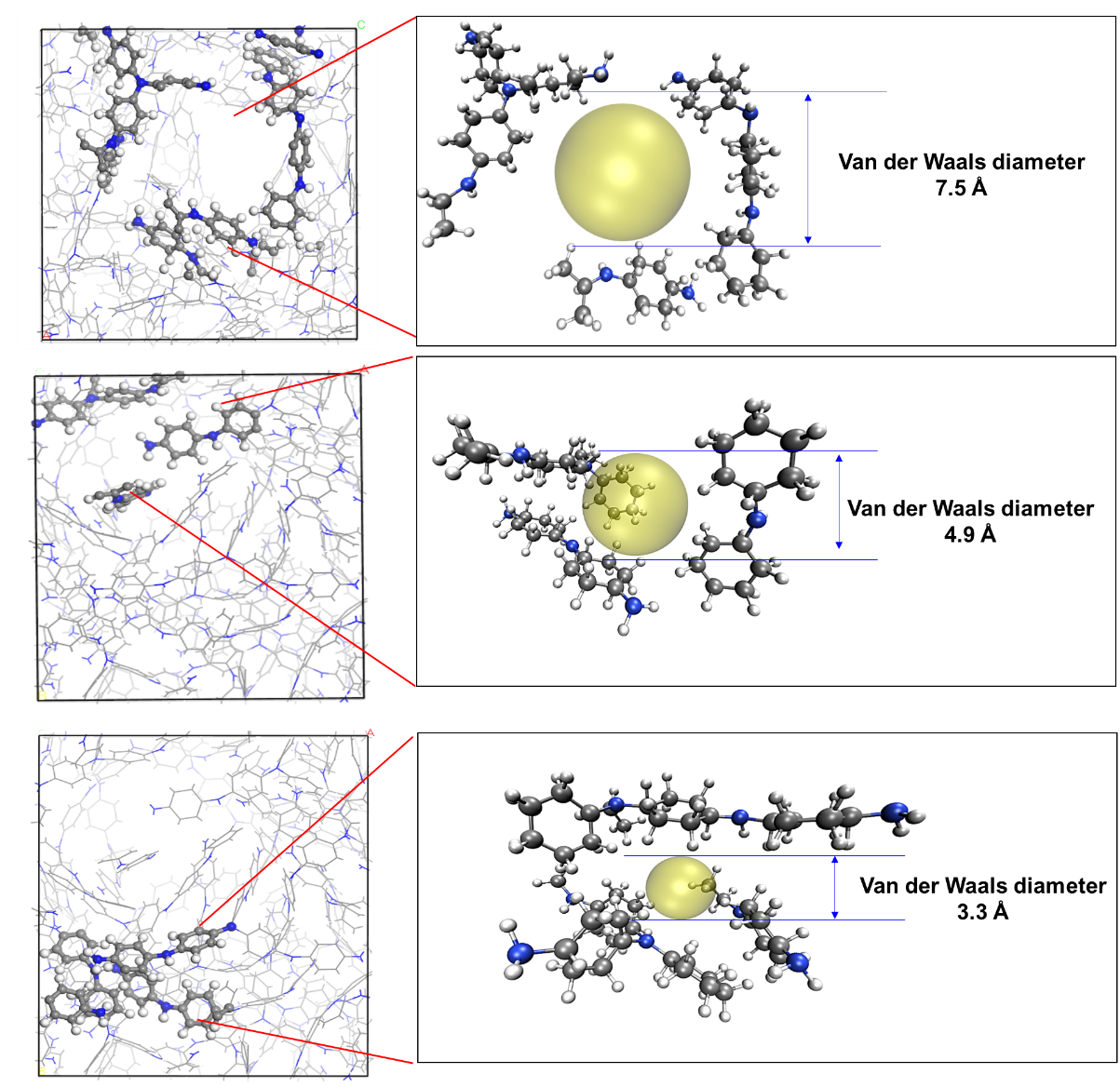


Figure S28. Three different confined fragmentation with pore size with van der Waals diameter of 7.5, 4.9 and 3.3 Å, respectively.


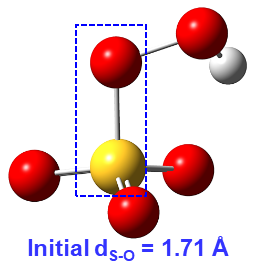


Figure S29. Optimized PMS structure using B3LYP/CC-pCTZ basic set.


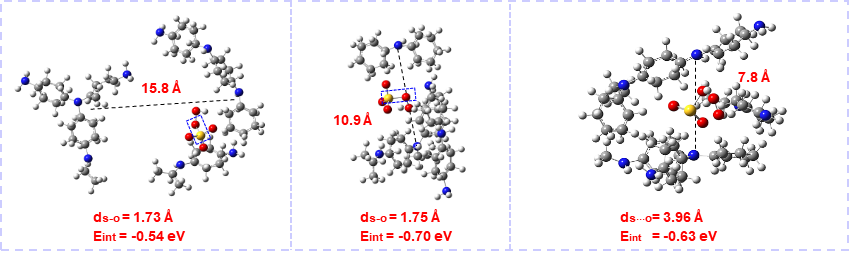


Figure S30. Energetic and geometric data of PTPA and PMS under different pore sizes.


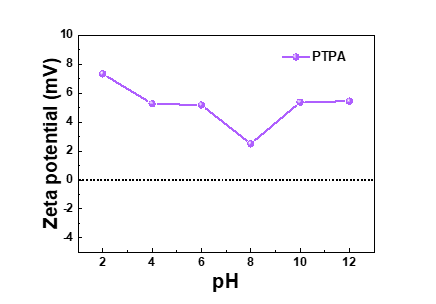


Figure S31. Zeta potential values of PTPA.


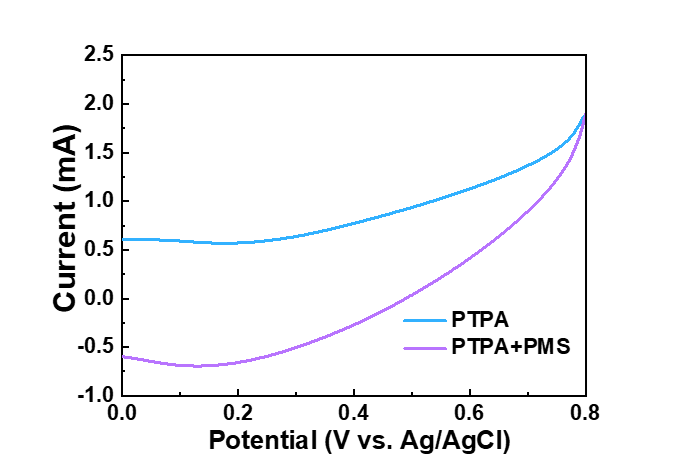


Figure S32. LSV curves of PTPA.

Based on the above analyses, a possible degradation mechanism is proposed as follows (Equations (S5-S10)). The HSO_5_^—^ is first adsorbed on the N atoms of PTPA in the non-confined space by the electrostatic interaction owing to the positive potential of PTPA (**Figure S31**), and then =NR^+^-/=N- received an electron from PMS to convert into -NR_2_^+^-/-NR-, where O_2_^•—^ is released (Equation (S5)) (minority).^[7]^ The process of electron transfer from PMS to PTPA was supported by an obviously increase in current for the PTPA electrode in the presence of PMS in the linear sweep voltammetry (LSV) curves (**Figure S32**).^[8]^ Then part of ^1^O_2_ is generated from the intermediate of O_2_^•—^ (Equations (S6-S8)) (minority).^[9]^ Besides, the majority of ^1^O_2_ is yielded from the dissociation of PMS under angstrom-confinement space *via* the cleavage of S-O and the subsequent proton transfer process (Equations (S9-S10)) (majority).

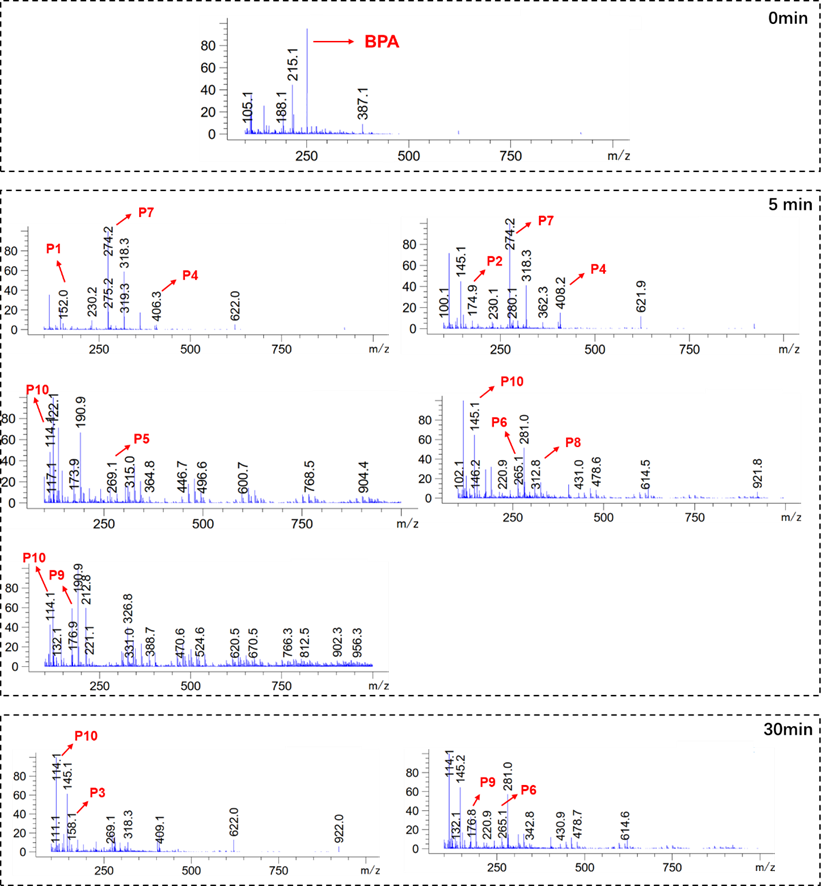


Figure S33. BPA degradation intermediates ([P+Na]^+^) in the PTPA/PMS system.


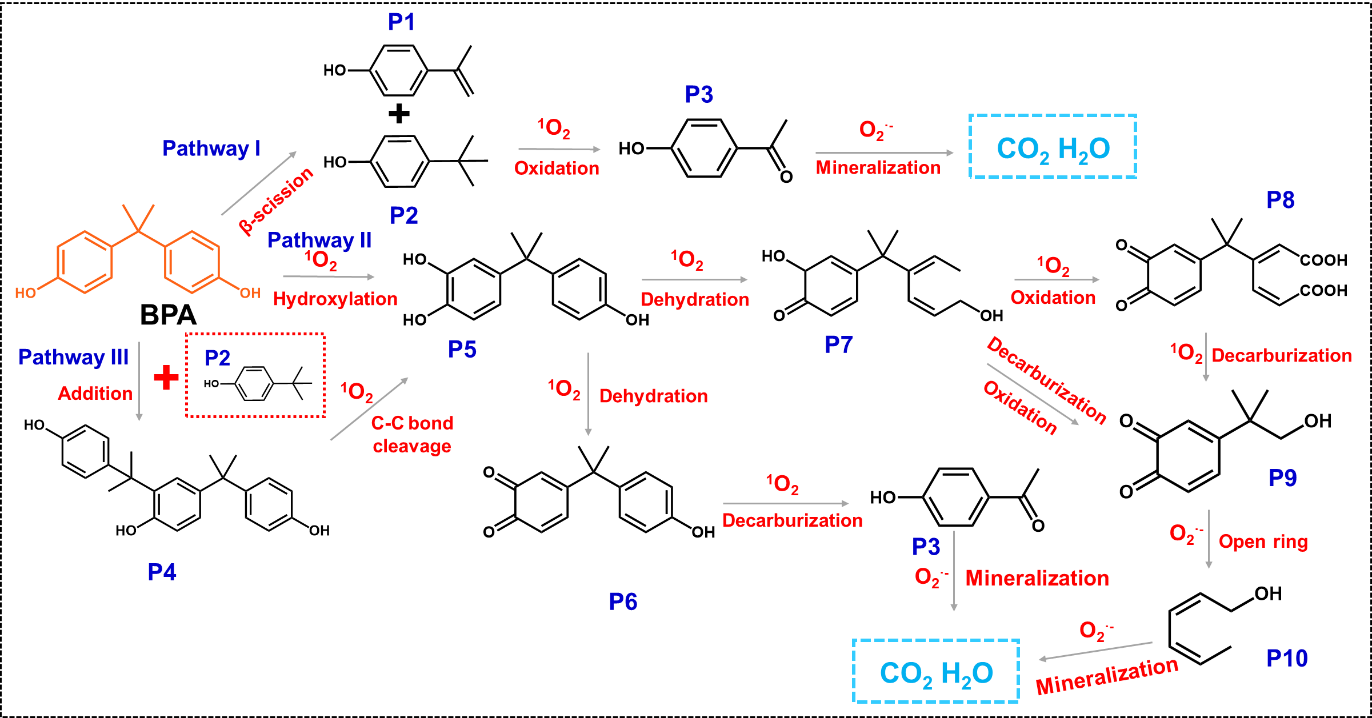


Figure S34. Degradation pathways of BPA degradation by catalytic PMS using PTPA.

The degradation intermediates of BPA by the PTPA/PMS system were identified by liquid chromatography-mass spectrometry (LC-MS). **Figure S33** shows the possible intermediate products in the BPA degradation at different time, and the possible degradation pathway is proposed (**Figure S34**). As can be seen in pathway I, the degradation was initiated by β-scission of isopropyl between two phenyl groups in BPA when attacked by ^1^O_2_, resulting in P1 and P2. Then P1 was gradually oxidized to P3,^[10]^ and finally converted into CO_2_ and H_2_O by O_2_^•—^. In pathway II, BPA was transformed to P5 by the hydroxylation in the presence of ^1^O_2_.^[11]^ Then the hydroxylated BPA was dehydrated to form P6 and P7.^[12]^ After that, the benzene ring was oxidized to form carboxylic acid and dicarboxylic acid by ^1^O_2_ and then decarburized into P3, P8 and P9.^[13]^ Subsequently, the chemical bond in P9 was broken under the attack of O_2_^•—^ and transformed into P10, which was finally converted into CO_2_ and H_2_O.^[14]^ In pathway III, BPA may bind with P2 to form compound P4, then the chemical bond between benzene ring and butyl was destroyed and formed P5.^[15]^ And it can be converted into CO_2_ and H_2_O after further oxidation and ring opening.^[16]^


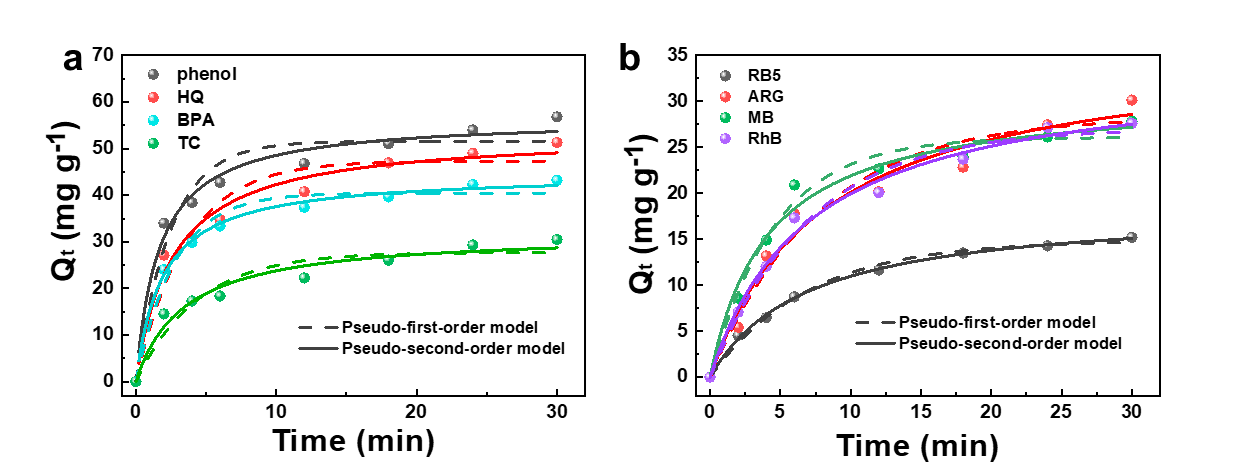


Figure S35. Adsorption efficiency of PTPA for organic pollutants with different molecular sizes. (Conditions: [Catalyst] = 0.2 g L^-1^, [pollutants] = 25 mg L^-1^, T = 25 °C, initial pH 6.0)

**
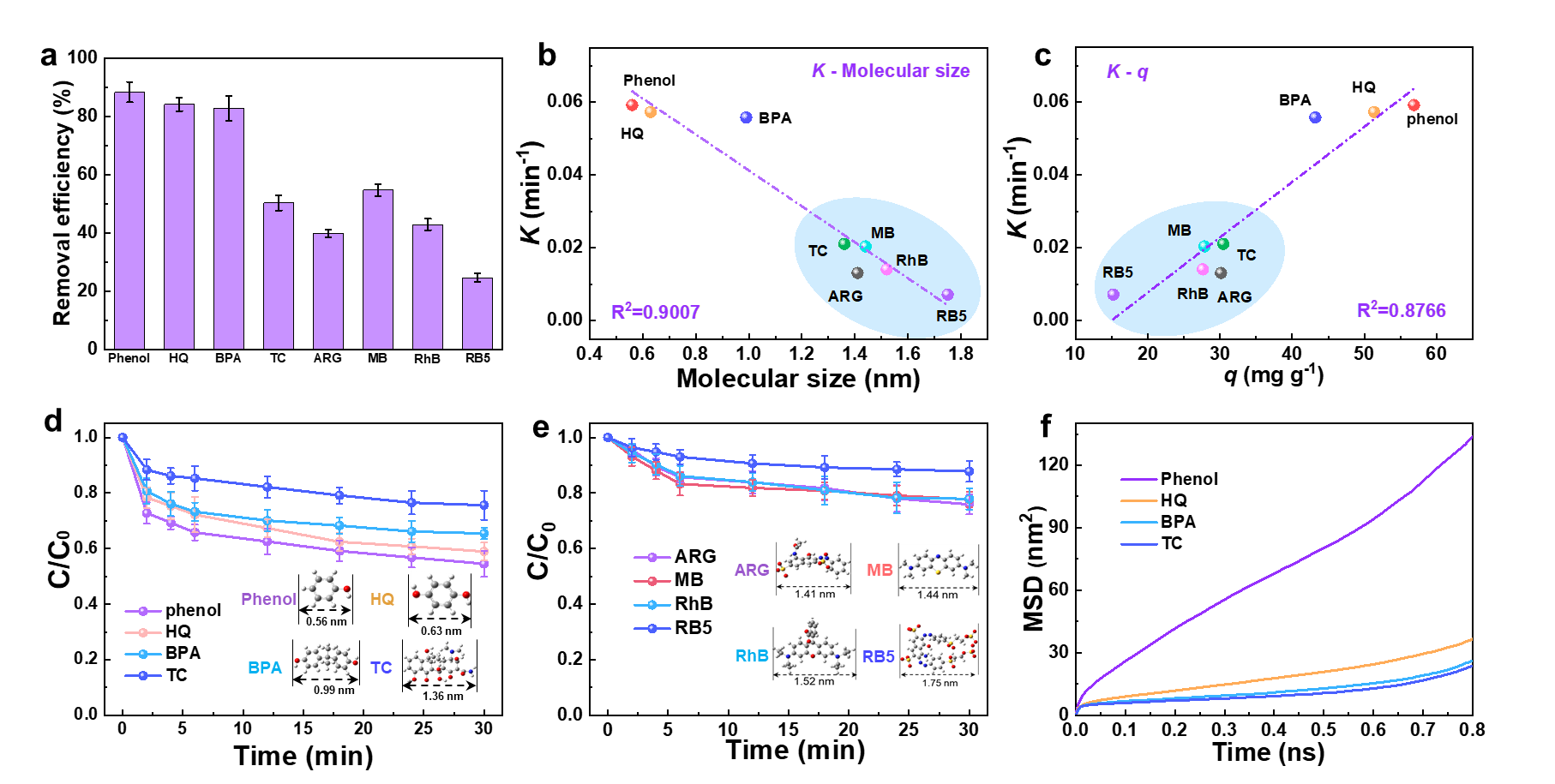
**

Figure S36. Removal efficiency of organic pollutants with different sizes by PTPA/PMS system. (a) Removal efficiency of organic pollutants with different size under PTPA/PMS system (Conditions: [Catalyst] = 0.2 g L^-1^, [BPA] = [Phenol] = [HQ] = [TC] = 25 mg L^-1^, [PMS] = 1.0 mmol L^-1^, T = 25 ^o^C, initial pH 6.0); (b) correlations of *K_obs_* and molecular size; (c) correlations of *K_obs_* and adsorption capacity; (d) and (e) adsorption efficiency of organic pollutants with different size; (f) mean-squared displacement (MSD) of various pollutants through PTPA.

Surprisingly, we found the unique sieving effect of this confinement system for selectively degrading targeted pollutants with different sizes. Four electron-rich compounds with similar molecular structures (phenolic hydroxyl) (phenol, HQ, BPA and TC),^[9, 17]^ but with different molecular sizes were chosen (**Figure S36a**). We show that phenol with molecular size of 0.56 nm could be rapidly degraded with a highest *K* value of 5.92×10^-2^ min^-1^ (**Figure S36b**). It should be noted that the rate constant (3.8±5.5×10^7^ M^-1^ s^-1^) for the reaction between ^1^O_2_ and HQ is higher than that (2.6±4.0×10^6^ M^-1^ s^-1^) between ^1^O_2_ and phenol.^[18]^ The lower degradation efficiency and *K* value for HQ may be related to its larger molecular size when compared with phenol. Moreover, a lowest *K* value of 2.11×10^-2^ min^-1^ and only 50% removal efficiency were obtained for TC, which can be easily attacked by ^1^O_2_.^[17d]^ Such a low removal efficiency in the PTPA/PMS system may be due to the fact that the molecular size of 1.36 nm of TC is larger than the mainly micropore size of PTPA (0.58 nm). Besides, the catalytic degradation efficiency of other different compounds (*i.e.*, anionic dyes including RB5 and ARG; cationic dyes including MB and RhB) with the molecular size larger than 1.40 nm are less than 60% (**Figure S36a**). The *K_obs_* follows the order of *K*_Phenol_ > *K*_HQ_ > *K*_BPA_ > *K*_TC_ > *K*_MB_ > *K*_RhB_ > *K*_ARG_ > *K*_RB5_ (**Figure S36b**). Further adsorption studies of PTPA on these pollutants show that *K*_obs_ is positively correlated with adsorption capacity (*q*) (**Figure S36c** and **Table S5**), indicating the adsorption-related removal feature of the PTPA/PMS system. Given the short lifetime (2~4 μs) of ^1^O_2_ and short diffusion distance (~220 nm at 4 μs),^[19]^ we deduce that the adsorption of pollutants by the micropores accelerated the enrichment of pollutants on the surface of catalysts, resulting in the increase of reaction rate between ^1^O_2_ and pollutants. The obtained results reveal this difference in degradation performance may be resulted from the size sieving effect of the microporous of PTPA.


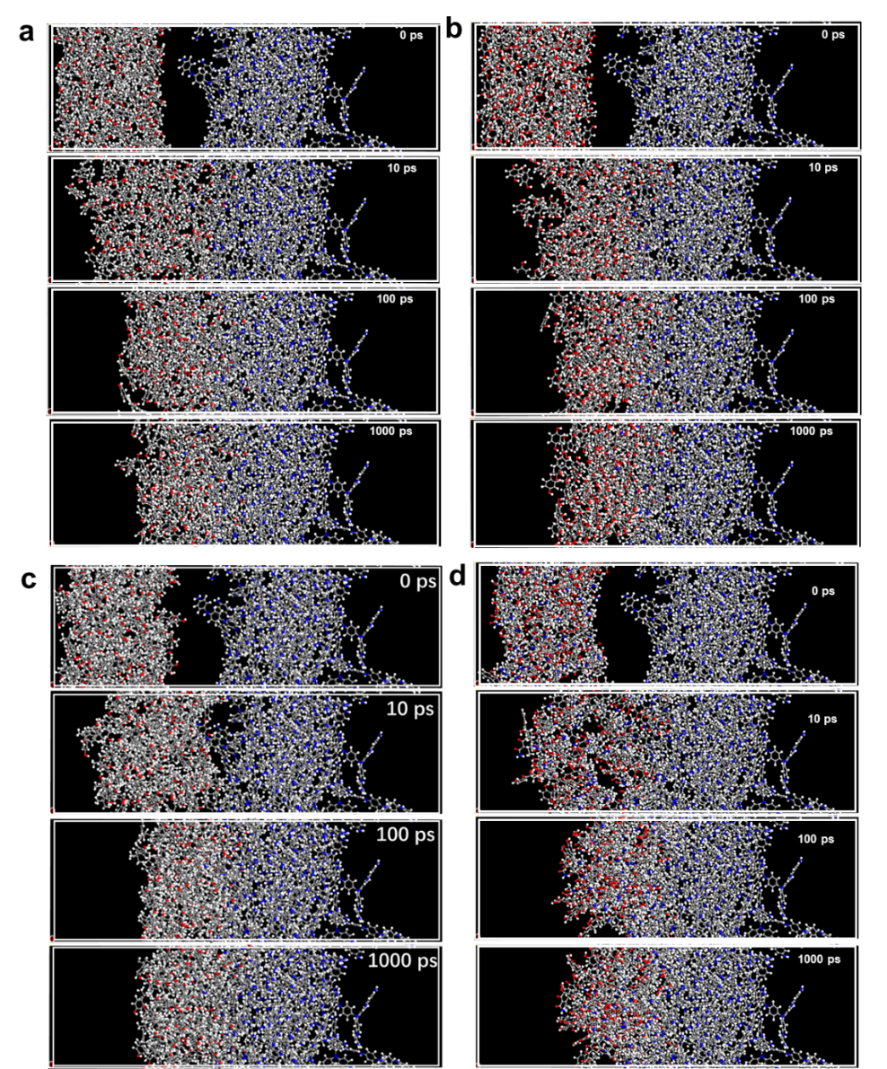


Figure S37. Simulation snapshots of (a) phenol, (b) HQ, (c) BPA and (d) TC molecules diffusing into the PTPA pore voids at 0 ps, 10 ps, 100 ps and 1000 ps.

We performed molecular simulations to help understand the diffusion behavior of four neutral pollutants into PTPA. Materials dynamic (MD) simulations verify that molecules with small size can rapidly diffuse into the voids of PTPA (**Figure S36f** and **S37**). It should be noted that the unobvious difference between the diffusion behaviour of BPA and TC may be due to the smaller void size of PTPA model than that of the practical one. Nevertheless, the difference in degradation performance between them suggests the occurrence of size sieving effect, where TC was difficulty to diffuse into PTPA microporous channels to be attacked by ^1^O_2_.

**
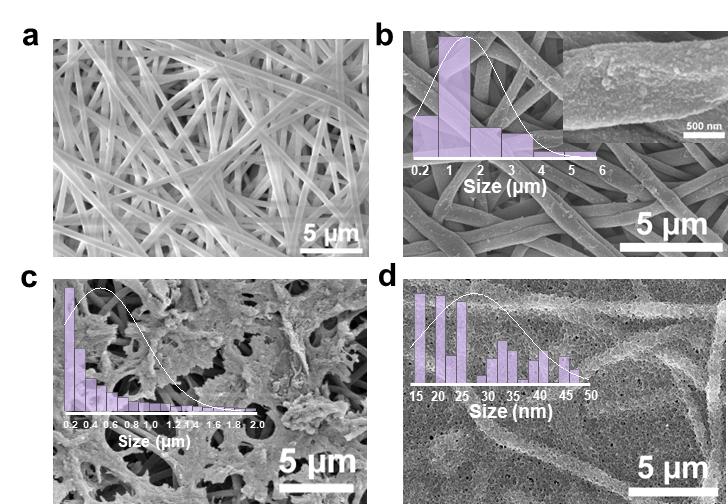
**

Figure S38. SEM images of (a) PAN-NFM, (b) PTPA@PAN-NFM, (c) PPTPA@PAN-NFM and (d) BPTPA@PAN-NFM.


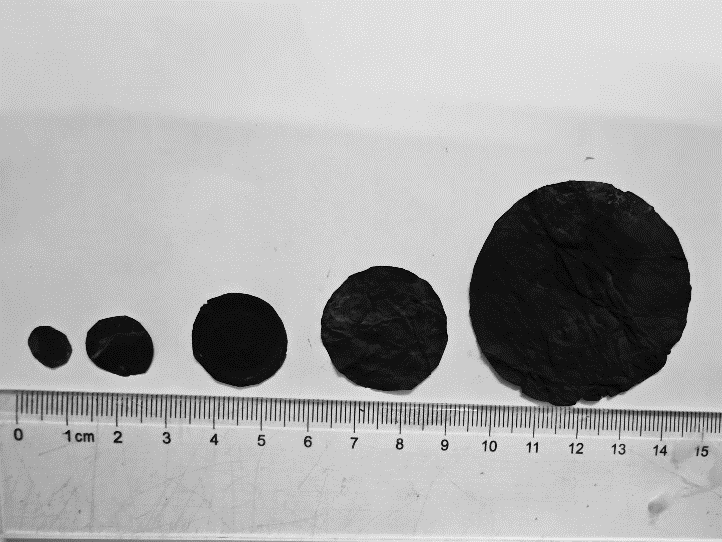


Figure S39. The photographs of BPTPA@PAN-NFM with different sizes.


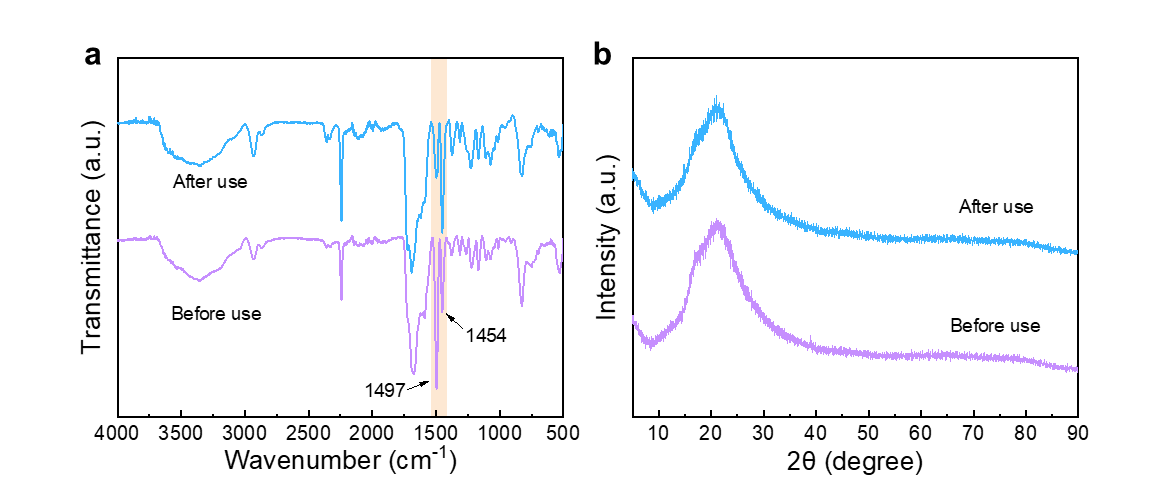


Figure S40. (a) FT-IR spectra and (b) XRD patterns of BPTPA@PAN-NFM before and after continuous-flow operation (120 h).


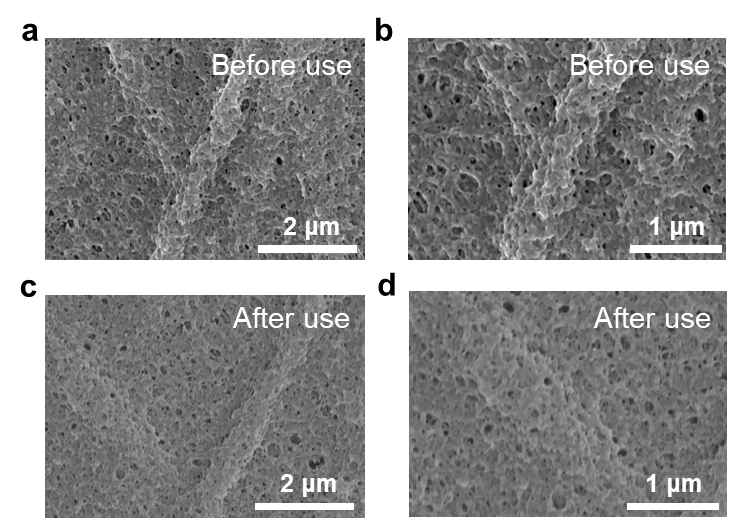


Figure S41. SEM images of BPTPA@PAN-NFM before (a-b) and after (c-d) 120 h continuous-flow operation (120 h).


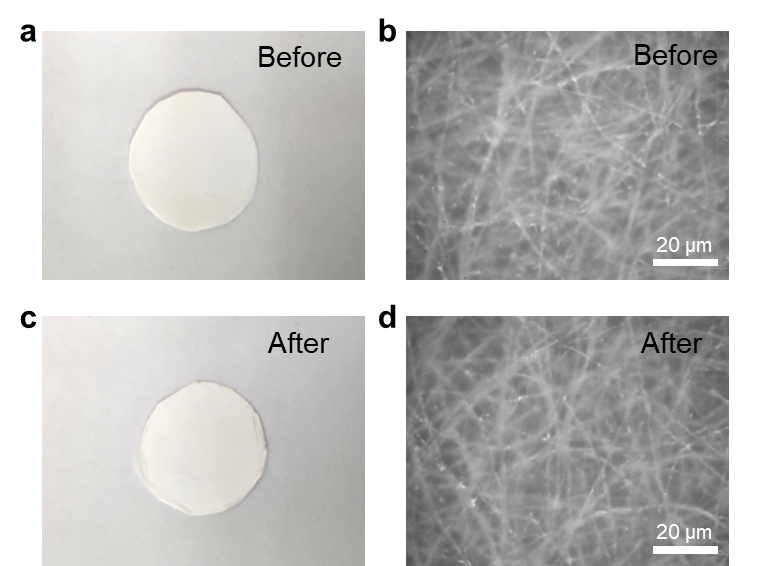


Figure S42. The photographs and microscope images of the support membrane before (a-b) and after (c-d) continuous-flow operation (120 h).


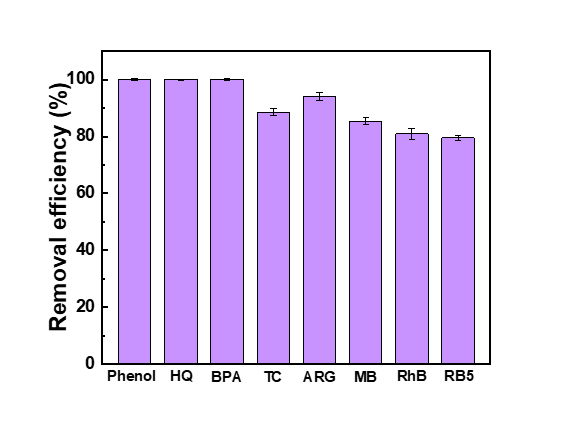


Figure S43. Removal efficiency of different pollutants in the BPTPA@PAN-NFM/PMS system.

**
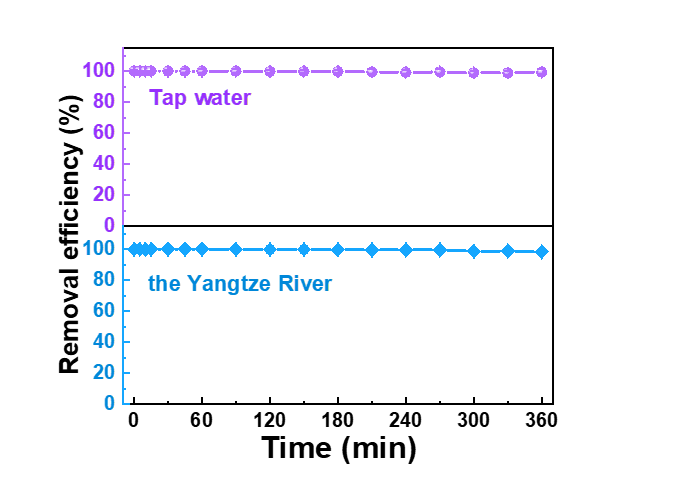
**

Figure S44. Influence of tap water and the Yangtze River on pollutant removal in the BPTPA@PAN-NFM/PMS system.

**
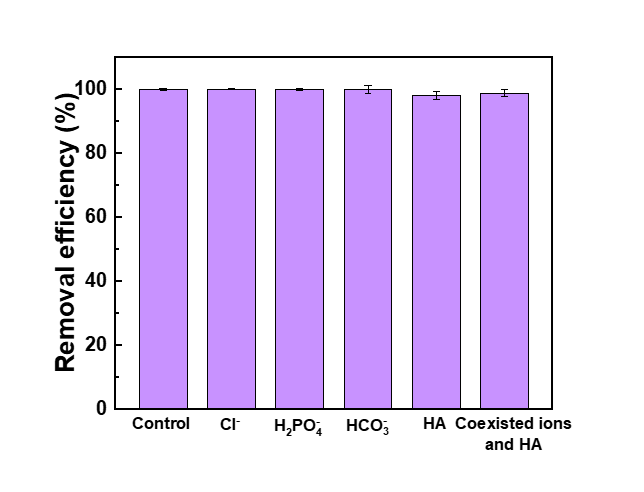
**

Figure S45. The effect of different anions, HA, and coexisted ions and HA on the BPA removal efficiency in the BPTPA@PAN-NFM/PMS system.

**
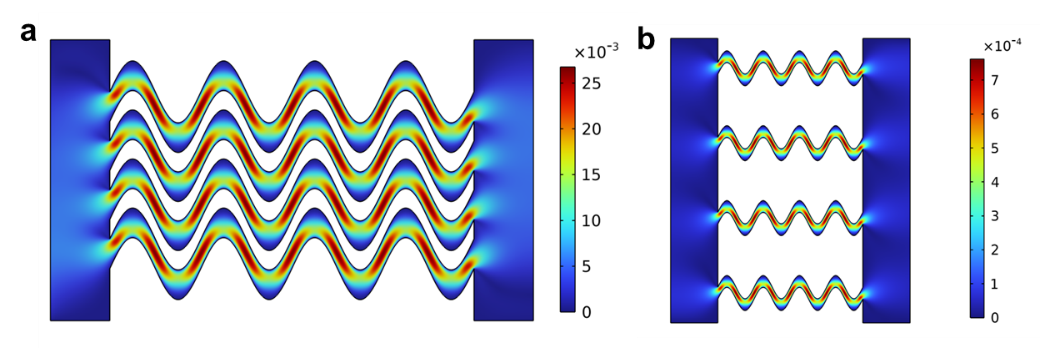
**

Figure S46. The diffusion velocity of liquid on mesopores among (a) ma-c and (b) me-c.


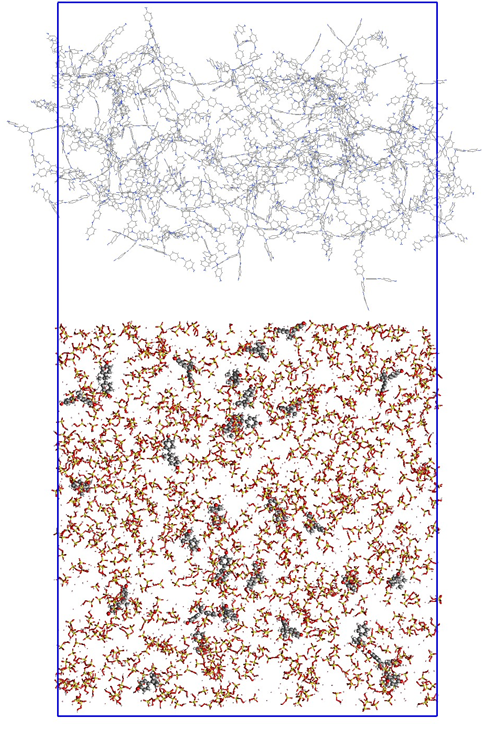


Figure S47. The distribution of BPA molecules at 0 ns for the diffusion system.

Supplementary Tables

Table S1. Elemental analysis of PTPA and PANI.

| **Sample** | **C /%** | **H /%** | **N /%** |
| --- | --- | --- | --- |
| **PTPA** | 71.76 | 4.78 | 8.89 |
| **PANI** | 50.70 | 4.37 | 9.81 |
| **PTPA-C^[a]^** | 68.23 | 4.84 | 7.92 |

[a] PTPA-C is the sample after using for PMS’s activation.

Table S2. Parameters of pore structures of samples by N_2_ adsorption isotherms.

| **Sample** | **S_BET_ ^[^**^a]^  **m^2^/g** | **S_micro_ ^[^**^b]^  **m^2^/g** | **V_micro_ ^[^**^c]^  **cc/g** | **V_T_ ^[^**^d]^  **cc/g** |
| --- | --- | --- | --- | --- |
| **PANI** | 16 | 3 | 0.002 | 0.029 |
| **PANI-C** | 43 | 9 | 0.005 | 0.073 |
| **PTPA** | 684 | 301 | 0.183 | 0.496 |
| **PTPA-0** | 15 | 9 | 0.005 | 0.016 |

[a] determined by the standard Brunauer–Emmett–Teller method; [b], [c]determined by t-plot method; [d] determined at a pressure of p/p_0_ = 0.95.

Table S3. Comparison between PTPA and the recently reported metal-free catalysts in the catalytic degradation activity on BPA.

| **System** | **Conditions** | **t**  **(min)** | **K_obs_**  **(**$\boldsymbol{\times}$ **10^-2^ min^-1^)** | **Mechanism** | **BET**  **surface area (m^2^/g)** | **Ref.** |
| --- | --- | --- | --- | --- | --- | --- |
| **PI-gC_3_N_4_/PMS** | Catalyst=1 g L^-1^  PMS=5 mM  BPA=10 mg L^-1^ | 60 | 5.01 | •OH, SO_4_^•−^,  O_2_^•−^, ^1^O_2_,  h^+^ | 22 | ^[20]^ |
| **CNS/PMS/Vis** | Catalyst=0.3 g L^-1^  PMS= 0.3 g L^-1^  BPA=50 mg L^-1^ | 120 | 1.31 | •OH, h^+^ | 86.8 | ^[21]^ |
| **COF-PRD/PMS/VL** | Catalyst=0.3 g L^-1^  PMS= 0.5 g L^-1^  BPA=10 mg L^-1^ | 150 | 2.1 | SO_4_^•−^, ^1^O_2_,  h^+^ | 206 | ^[22]^ |
| **PCNC/Vis-PMS** | Catalyst=0.33 g L^-1^  PMS= 0.03 g L^-1^  BPA=20 mg L^-1^ | 30 | 9.8 | SO_4_^•−^, O_2_^•−^,  ^1^O_2_, h^+^ | 150 | ^[23]^ |
| **CNGO-3/PMS/light** | Catalyst=0.4 g L^-1^  PMS= 0.8 mM  BPA=10 mg L^-1^ | 60 | 6.19 | •OH, SO_4_^•−^ | 16 | ^[24]^ |
| **ZIF-NC/g-C_3_N_4_/Vis-PMS** | PMS= 2 mM  BPA=20 mg L^-1^ | 60 | 5.13 | SO_4_^•−^, O_2_^•−^,  •OH, h^+^ | / | ^[25]^ |
| **CN-AA_0.3_/PMS** | Catalyst=0.5 g L^-1^  PMS= 5mM  BPA=23 mg L^-1^ | 60 | 9.21 | •OH, SO_4_^•−^,  ^1^O_2_ | 127.76 | ^[26]^ |
| **NCNs-9/PDS** | Catalyst=0.12 g L^-1^  PDS= 3.25 mM  BPA=20 mg L^-1^ | 20 | 12.6 | SO_4_^•−^, O_2_^•−^,  •OH, ^1^O_2_ | 565.72 | ^[27]^ |
| **PTPA/PMS** | Catalyst=0.25 g L^-1^  PMS= 1 mM  BPA=25 mg L^-1^ | 30 | 6.09 | ^1^O_2_ (98.7%), O_2_^•−^ | 685 | **This work** |

Table S4. The effect of different anions, HA, and coexisted ions and HA on BPA degradation in the PTPA/PMS system.

| **Influence parameter** | **Concentration** | **K_obs_ (**$\times$ **10^-2^ min^-1^)** | **Removal (%)** |
| --- | --- | --- | --- |
| **Control** | / | 5.58 | 87.3 |
| **Cl^—^** | 5 mmol L^-1^ | 6.88 | 91.4 |
|  | 10 mmol L^-1^ | 8.39 | 95.7 |
|  | 15 mmol L^-1^ | 10.16 | 97.7 |
| **H_2_PO_4_^—^** | 5 mmol L^-1^ | 5.05 | 87.5 |
|  | 10 mmol L^-1^ | 5.52 | 88.2 |
|  | 15 mmol L^-1^ | 5.80 | 89.9 |
| **HCO_3_^—^** | 5 mmol L^-1^ | 5.73 | 88.7 |
|  | 10 mmol L^-1^ | 5.97 | 89.4 |
|  | 15 mmol L^-1^ | 7.16 | 93.5 |
| **HA** | 5 mg L^-1^ | 4.61 | 80.9 |
|  | 10 mg L^-1^ | 4.19 | 78.6 |
|  | 15 mg L^-1^ | 4.16 | 74.2 |
| **Coexisted ions and HA** | Cl^—^ (15 mmol L^-1^) | 6.03 | 90.1 |
|  | H_2_PO_4_^—^ (15 mmol L^-1^) |  |  |
|  | HCO_3_^—^ (15 mmol L^-1^) |  |  |
|  | HA (15 mg L^-1^) |  |  |

Table S5. Kinetic parameters of adsorption of organic pollutants with different molecular sizes on PTPA.

| **Pollutant** | ***Q_e,exp_* /**  **mg g^-1^** | **Pseudo-first-order model** | | | **Pseudo-second-order model** | | |
| --- | --- | --- | --- | --- | --- | --- | --- |
|  |  | ***K_1_*/**  **min^-1^** | ***Q_e,cal_* /**  **mg g^-1^** | ***R*^2^** | ***K*_2_ / g mg^-1^ min^-1^** | ***Q_e,cal_* /**  **mg g^-1^** | ***R*^2^** |
| **Phenol** | 56.85 | 0.4017 | 51.55 | 0.9445 | 0.0106 | 56.63 | 0.8999 |
| **HQ** | 51.35 | 0.2815 | 47.31 | 0.9366 | 0.0071 | 53.38 | 0.9117 |
| **BPA** | 43.19 | 0.3620 | 40.46 | 0.9708 | 0.0117 | 44.72 | 0.9760 |
| **TC** | 30.47 | 0.2294 | 27.73 | 0.9130 | 0.0090 | 31.99 | 0.8752 |
| **ARG** | 30.14 | 0.1307 | 28.35 | 0.9570 | 0.0034 | 36.24 | 0.9455 |
| **MB** | 27.86 | 0.2179 | 26.09 | 0.9782 | 0.0078 | 30.93 | 0.9516 |
| **RhB** | 27.61 | 0.1435 | 27.07 | 0.9809 | 0.0042 | 33.97 | 0.9790 |
| **RB5** | 15.22 | 0.1447 | 14.85 | 0.9920 | 0.0078 | 18.56 | 0.9963 |

Table S6. The structural characteristics and removal performance of PTPA@PAN-NFM, PPTPA@PAN-NFM and BPTPA@PAN-NFM membranes.

| **Sample** | **Loading amount (mg cm^-2^)** | **Membrane surface’s structure** | **Hierarchical channels** | **Flux (L m^-2^ h^-1^ bar^-1^)** | **Removal efficiency** |
| --- | --- | --- | --- | --- | --- |
| **PTPA@PAN-NFM** | 0.37 | Macropores and micropores | Macropores-micropores | 3303 | 42.1% |
| **PPTPA@PAN-NFM** | 0.45 | Macropores, mesopores and micropores | Macropores-mesopores-micropores | 3025 | 67.7% |
| **BPTPA@PAN-NFM** | 0.53 | Mesopores and micropores | Macropores-mesopores-micropores | 2870 | 100.0% |

References

[1] X. Zhou, C. Luo, M. Luo, Q. Wang, J. Wang, Z. Liao, Z. Chen, Z. Chen, *Chem. Eng. J.* **2020**, *381*, 122587.

[2] V. M. Suresh, S. Bonakala, S. Roy, S. Balasubramanian, T. K. Maji, *J. Phys. Chem. C* **2014**, *118*, 24369.

[3] S. Plimpton, *J. Comput. Phys.* **1995**, *117*, 1.

[4] M. J. Frisch, G. W. Trucks, H. B. Schlegel, G. E. Scuseria, M. A. Robb, J. R. Cheeseman, G. Scalmani, V. Barone, B. Mennucci, G. A. Petersson, H. Nakatsuji, M. Caricato, X. Li, H. P. Hratchian, A. F. Izmaylov, J. Bloino, G. Zheng, J. L. Sonnenberg, M. Hada, M. Ehara, K. Toyota, R. Fukuda, J. Hasegawa, M. Ishida, T. Nakajima, Y. Honda, O. Kitao, H. Nakai, T. Vreven, J. A. Montgomery Jr., J. E. Peralta, F. Ogliaro, M. J. Bearpark, J. Heyd, E. N. Brothers, K. N. Kudin, V. N. Staroverov, R. Kobayashi, J. Normand, K. Raghavachari, A. P. Rendell, J. C. Burant, S. S. Iyengar, J. Tomasi, M. Cossi, N. Rega, N. J. Millam, M. Klene, J. E. Knox, J. B. Cross, V. Bakken, C. Adamo, J. Jaramillo, R. Gomperts, R. E. Stratmann, O. Yazyev, A. J. Austin, R. Cammi, C. Pomelli, J. W. Ochterski, R. L. Martin, K. Morokuma, V. G. Zakrzewski, G. A. Voth, P. Salvador, J. J. Dannenberg, S. Dapprich, A. D. Daniels, Ö. Farkas, J. B. Foresman, J. V. Ortiz, J. Cioslowski, D. J. Fox, Gaussian, Inc., Wallingford, CT, USA **2009**.

[5] L. Sarkisov, A. Harrison, *Mol. Simul.* **2011**, *37*, 1248.

[6] B. Liang, H. Wang, X. Shi, B. Shen, X. He, Z. A. Ghazi, N. A. Khan, H. Sin, A. M. Khattak, L. Li, *Nat. Chem.* **2018**, *10*, 961.

[7] W. Duan, J. He, Z. Wei, Z. Dai, C. Feng, *Environ. Sci.: Nano* **2020**, *7*, 2982.

[8] L. S. Zhang, X. H. Jiang, Z. A. Zhong, L. Tian, Q. Sun, Y. T. Cui, X. Lu, J. P. Zou, S. L. Luo, *Angew. Chem. Int. Ed.* **2021**, *60*, 21751.

[9] X. Tian, P. Gao, Y. Nie, C. Yang, Z. Zhou, Y. Li, Y. Wang, *Chem. Comm.* **2017**, *53*, 6589.

[10] S. Wang, J. Tian, Q. Wang, F. Xiao, S. Gao, W. Shi, F. Cui, *Appl. Catal. B* **2019**, *256*, 117783.

[11] D. Guo, Y. Yao, S. You, L. Jin, P. Lu, Y. Liu, *Appl. Catal. B* **2022**, *309*, 121289.

[12] M. Zhang, C. Xiao, X. Yan, S. Chen, C. Wang, R. Luo, J. Qi, X. Sun, L. Wang, J. Li, *Environ. Sci. Technol.* **2020**, *54*, 10289.

[13] C. Li, S. Yang, R. Bian, Y. Tan, X. Zhang, S. Zheng, Z. Sun, *Chem. Eng. J.* **2022**, *448*, 137746.

[14] Z. Lyu, M. Xu, J. Wang, A. Li, P. F. X. Corvini, *Chem. Eng. J.* **2022**, *433*, 133581.

[15] C. Li, S. Yang, R. Bian, Y. Tan, X. Dong, N. Zhu, X. He, S. Zheng, Z. Sun, *J. Hazard. Mater.* **2021**, *407*, 124736.

[16] Y. Tan, C. Li, Z. Sun, R. Bian, X. Dong, X. Zhang, S. Zheng, *Chem. Eng. J.* **2020**, *388*, 124386.

[17] a)Y. Gao, T. Wu, C. Yang, C. Ma, Z. Zhao, Z. Wu, S. Cao, W. Geng, Y. Wang, Y. Yao, *Angew. Chem. Int. Ed.* **2021**, *60*, 22513; b)Q. Yang, Y. Yan, X. Yang, G. Liao, J. He, D. Wang, *Chem. Eng. J.* **2022**, *429*, 132178; c)X. Liu, Y. Liu, H. Qin, Z. Ye, X. Wei, W. Miao, D. Yang, S. Mao, *Environ. Sci. Technol.* **2022**, *56*, 2665; d)S. Li, Y. Yang, H. Zheng, Y. Zheng, C.-S. He, B. Lai, J. Ma, J. Nan, *Water Res.* **2022**, *225*, 119176.

[18] P. G. Tratnyek, J. Hoigne, *Environ. Sci. Technol.* **1991**, *25*, 1596.

[19] a) P. B. Merkel, D. R. Kearns, *J. Am. Chem. Soc.* **1972**, *94*, 1029; b) E. Skovsen, J. W. Snyder, J. D. Lambert, P. R. Ogilby, *J. Phys. Chem. B* **2005**, *109*, 8570; c) R. W. Redmond, I. E. Kochevar, *Photochem. Photobiol.* **2006**, *82*, 1178.

[20] J. Zhang, X. Zhao, Y. Wang, Y. Gong, D. Cao, M. Qiao, *Appl. Catal. B* **2018**, *237*, 976.

[21] K. Y. A. Lin, Z. Y. Zhang, *Chem. Eng. J.* **2017**, *313*, 1320.

[22] F. Liu, Q. Dong, C. Nie, Z. Li, B. Zhang, P. Han, W. Yang, M. Tong, *Chem. Eng. J.* **2022**, *430*, 132833.

[23] H. Ming, D. Wei, Y. Yang, B. Chen, C. Yang, J. Zhang, Y. Hou, *Chem. Eng. J.* **2021**, *424*, 130296.

[24] P. Qiu, Z. Cheng, N. Xue, Y. Zeng, X. Kai, S. Zhang, C. Xu, F. Liu, Z. Guo, *Carbon* **2021**, *178*, 81.

[25] Y. Gong, X. Zhao, H. Zhang, B. Yang, K. Xiao, T. Guo, J. Zhang, H. Shao, Y. Wang, G. Yu, *Appl. Catal. B* **2018**, *233*, 35.

[26] Y. Zhu, Z. Chen, Y. Gao, C. Hu, *J. Hazard. Mater.* **2020**, *394*, 122578.

[27] H. Yin, F. Yao, Z. Pi, Y. Zhong, L. He, K. Hou, J. Fu, S. Chen, Z. Tao, D. Wang, I. *J. Colloid Interface Sci.* **2021**, *586*, 551.
